# Discovery of a potent small molecule inhibiting Huntington’s disease (HD) pathogenesis *via* targeting CAG repeats RNA and Poly Q protein

**DOI:** 10.1038/s41598-019-53410-z

**Published:** 2019-11-14

**Authors:** Eshan Khan, Subodh Kumar Mishra, Ribhav Mishra, Amit Mishra, Amit Kumar

**Affiliations:** 1 Discipline of Biosciences and Biomedical Engineering, Indian Institute of Technology Indore, Simrol, Indore, 453552 India; 2 Cellular and Molecular Neurobiology Unit, Indian Institute of Technology Jodhpur, Rajasthan, 342011 India

**Keywords:** Biophysical chemistry, Data mining

## Abstract

CAG repeats RNA causes various fatal neurodegenerative diseases exemplified by Huntington’s disease (HD) and several spinocerebellar ataxias (SCAs). Although there are differences in the pathogenic mechanisms, these diseases share the common cause, i.e., expansion of CAG repeats. The shared cause of these diseases raises the possibility for the exploiting the common target as a potential therapeutic approach. Oligonucleotide-based therapeutics are designed earlier with the help of the base pairing rule but are not very promiscuous, considering the nonspecific stimulation of the immune system and the poor cellular delivery. Therefore, small molecules-based therapeutics are preferred for targeting the repeats expansion disorders. Here, we have used the chemical similarity search approach to discern the small molecules that selectively target toxic CAG RNA. The lead compounds showed the specificity towards AA mismatch in biophysical studies including CD, ITC, and NMR spectroscopy and thus aided to forestall the polyQ mediated pathogenicity. Furthermore, the lead compounds also explicitly alleviate the polyQ mediated toxicity in HD cell models and patient-derived cells. These findings suggest that the lead compound could act as a chemical probe for AA mismatch containing RNA as well as plays a neuroprotective role in fatal neurodegenerative diseases like HD and SCAs.

## Introduction

Classically, RNA was considered merely a carrier of genetic information. But modern views have stated RNA as a profound tool to modulate the gene expression as well as other biological processes. Although, RNA has been considered undruggable with the small molecules outside of bacterial ribosome, due to lack of their higher affinity and specificity. However, the use of new biochemical screening methods and design based technologies have provided compelling progress in this field^1^. Trinucleotide repeats (TNRs) are present in human genes, with only a few known functions. However, the expansion in some repeats above the threshold limits results in more than 40 neurological disorders^2,3^. These expansions can occur either in the coding region, like Huntington disease or in the non-coding region such as Fragile X-associated Tremor Ataxia Syndrome (FXTAS). CAG repeats RNA alone is involved in most of the trinucleotide repeats disorders, including Huntington’s disease (HD) and different spinocerebellar ataxia (SCAs). CAG repeats expansion can occur in 5′UTR of the gene PPP2R2B that causes SCA type 12 whereas expansion in exon region causes other late-onset progressive neurodegenerative disorders like HD, spinal and bulbar muscular atrophy (SBMA), dentatorubral-pallidoluysian atrophy (DPLA) and several other SCAs.

Provided all possible reading frames, expanded CAG repeats could freely encode into three different kinds of amino acids (CAG, GCA and AGC code for poly Q, poly A, and poly S, respectively). Earlier, reports stated that only poly Q protein aggregates are responsible for the pathogenesis and therefore these diseases are also known as polyglutamine (poly Q) diseases^4^. However, other homo-polymeric proteins produced by sense and antisense repeat-associated non-ATG (RAN) translation were also reported for diseases progression^5^. The early age onset and the severity of the TNR disorders are associated with a large number of repeats present in the genes. HD is one of the well-known trinucleotide repeats disease that is caused by the expansion of CAG repeats in the first exon of the IT15 gene (Interesting Transcript 15). Generally, a healthy individual contains up to 36 CAG repeats, while the number of CAG repeats is increased by more than 40 repeats in the coding region of the huntingtin gene in the diseased condition^6^. The global prevalence of HD varies widely, with a very high incidence in Caucasian while lowest in the Asian population^7^. Unfortunately, there is no cure for these devastating disease till date.

CAG repeats expansion translates into polyglutamine which further forms the polyQ aggregates, a crucial pathological hallmark of HD patients. The transcript of expanded CAG repeats can also fold into the non-canonical three-dimensional duplex hairpin loop structure with non-stranded A-A mismatch^8,9^. The size and stability of the hairpin are dependent on the CAG repeat length^10^. The formation of duplex hairpins recruits various RNA binding proteins like muscleblind-like (MBNL), DICER, several transcription factors, and nucleolar protein nucleolin. The sequestration of these crucial proteins results in the loss of their normal functions that further leads to aberrant post-transcriptional regulation^11–13^. Recently, repeat-associated non-ATG (RAN) translation in r(CAG)^exp^ has also been studied where four different types of homo-polymeric proteins are encoded by mutant CAG RNA that further results into toxic protein aggregates inside the cells^14,15^. All these mutual pathogenic mechanisms lead to a toxic condition inside the neuronal cells.

Various strategies have been reported as an HD therapeutics, including protein aggregation inhibitors^16,17^ (2)-Epigallocatechin-3-Gallate (EGCG) has been studied as a poly Q protein inhibitor in HD^18^. However, HD pathology is not limited to poly Q protein aggregation, but the mutant RNA also plays a vital role in disease development. Hence, targeting the actual toxic RNA rather than poly Q aggregation may provide promising results for HD therapeutics development. Oligonucleotide and peptide-based approaches targeting the toxic CAG RNA has the limitation of the blood-brain barrier, allergic reactions, and poor absorption^19^. Conversely, small molecule-based therapeutic approaches have shown promising results. Several other groups, as well as we have reported small molecules targeting expanded CAG RNA and providing hope for HD drug development^20–23^. However, the high affinity and specificity of small molecules are prerequisite for the therapy as HD treatment needs a long-term continuous dose. To explore compounds with similar shape or chemistry with improved biological activity or pharmacodynamics & pharmacokinetic properties, herein, we have used chemical similarity approach to screen the NCI library with a previously reported molecule, Myricetin as a query molecule.

Interestingly, we have found some small molecules with a better affinity and less toxicity than Myricetin. We have assessed the selectivity of potential lead compounds using CD, UV melting, various gel-based assays, and NMR spectroscopy. Moreover, the compounds were also tested in HD cellular models, and patient-derived cells to assess the applicability of our decrees to the HD pathogenesis.

## Results and Discussion

The rationally designed small molecules for this toxic 5′CAG/3′GAC RNA could provide a valuable avenue as a therapeutic approach for these Trinucleotide repeat expansion diseases^22,24,25^. Therefore, we have used chemical similarity search to explore new potent small molecules that could be utilized to target the pathogenic CAG repeats, causing HD and SCAs.

### Chemical Similarity search for Myricetin similar compounds

The 3D shape of small molecules is an important determinant of activity and functions^26,27^. Recently, shape-based small molecule screening has been considered as a promising tool in drug discovery because it encouragingly provides new molecules with better affinity and selectivity^28,29^. Herein, we have performed the chemical similarity approach and collected small molecules from the National Cancer Institute (NCI) with features that strongly predispose them to bind with RNA. The similar shape and structure of the molecules help it to bind within the same pocket and concurrently, exhibited similar biological activates. NCI database is an easily accessible database that contains more than 250,000 compounds with broad chemical spaces along with complete stereochemistry specifications. Rapid Overlay of Chemical Structures (ROCS)^30^ software uses 3D shape algorithm for 3D shape comparison between two molecules. The Gaussian function of ROCS helps to fasten the 3D shape-based calculations due to the involvement of maximal intersections of the volumes of two molecules. The compounds with complete stereochemistry specification and reliable 3D coordinates of stereoisomers were chosen for screening. The chemical similarity search of the compounds was done using Omega 2.3.2 (v 2.02) software^31^ from OpenEye Scientific Software. Chemical similarity search uses two different matrices: Shape Tanimoto coefficient^29^ for 3D similarities and color score^32^ for 3D chemistry alignment. Colour force field (implicit Mills–Dean)^32^ helps to align hydrogen bond donor, acceptor, a hydrophobic group, cations, anions, and rings. The similarity of two compounds is related to either shape or color score that lies in between 0 to 1, which states no or complete similarity respectively. Subsets of the top 19 compounds (Supplementary Table 1) based on similarity with previously reported molecule Myricetin^23^, were obtained from NCI and used for affinity-based screening against 1 × 1 loop 5′CAG/3′GAC RNA.

### Primary screening of small molecules using Fluorescence titration study

Several studies have shown that planar compounds are very efficient to bind with RNA motifs; notably, 1 × 1 nucleotide internal loop^22,23,33,34^. We and others have reported some of the natural and synthetic compounds binding with 1 × 1 loop 5′CAG/3′GAC RNA^23,35–37^.

**Figure 1:**
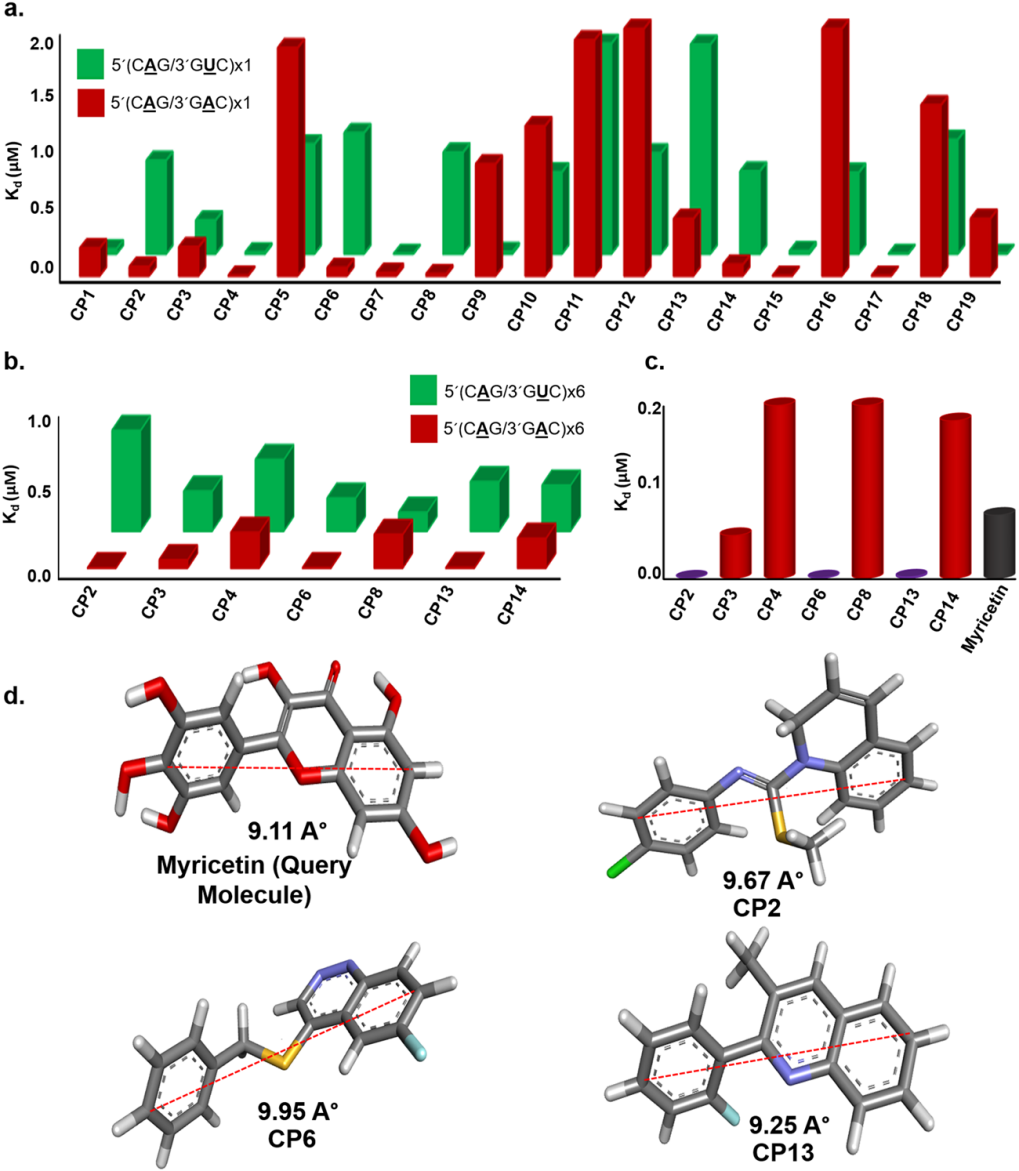
Screening of small molecules targeting CAG repeat RNA. (**a**) A plot of binding constant values of compounds against 1 × 1 single loop 5′CAG/3′GAC RNA with control duplex 5′CAG/3′GUC RNA (**b**) A plot of binding constant values of shortlisted compounds with multiple loop 5′CAG/3′GAC RNA (**c**) A comparative Plot of binding constant values of potential lead compounds with Myricetin (**d**) Structural analysis of lead compound in comparison to query molecule (Myricetin).

Compounds were further screened using the fluorescence titration study, against 1 × 1 loop 5′CAG/3′GAC and a control 5′CAG/3′GUC RNA. The codes were assigned to each of the compounds for better understanding (Supplementary Table 1). Interestingly, we have found some compounds that possess higher affinity for the 5′CAG/3′GAC motif RNA than Myricetin (Fig. 1, Supplementary Fig. 1, & Supplementary Table S2). The compounds that harbor comparatively less affinity for 1 × 1 loop 5′CAG/3′GAC RNA than control duplex 5′CAG/3′GUC RNA were not considered for further studies (Fig. 1a). Rest 7 compounds with high-affinity for 1 × 1 loop 5′CAG/3′GAC RNA was proceeded for further screening with (5′CAG/3′GAC)x6 loop RNA (Fig. 1b) using fluorescence titration study. Out of 7 compounds, only 3 compounds showed a higher affinity for (5′CAG/3′GAC)x6 RNA as compared to our previously reported compound Myricetin (Fig. 1c, Supplementary Table S3)^23^. Moreover, these 3 compounds obtained from primary screening showed almost similar distances between two distant atoms as presented in Myricetin which further affirms our chemical similarity-based approaches and strengthens the notion of exhibiting the similar biological activity (Fig. 1d).

### Secondary screening of small molecules against target RNA using Isothermal Titration Calorimetry

**Figure 2:**
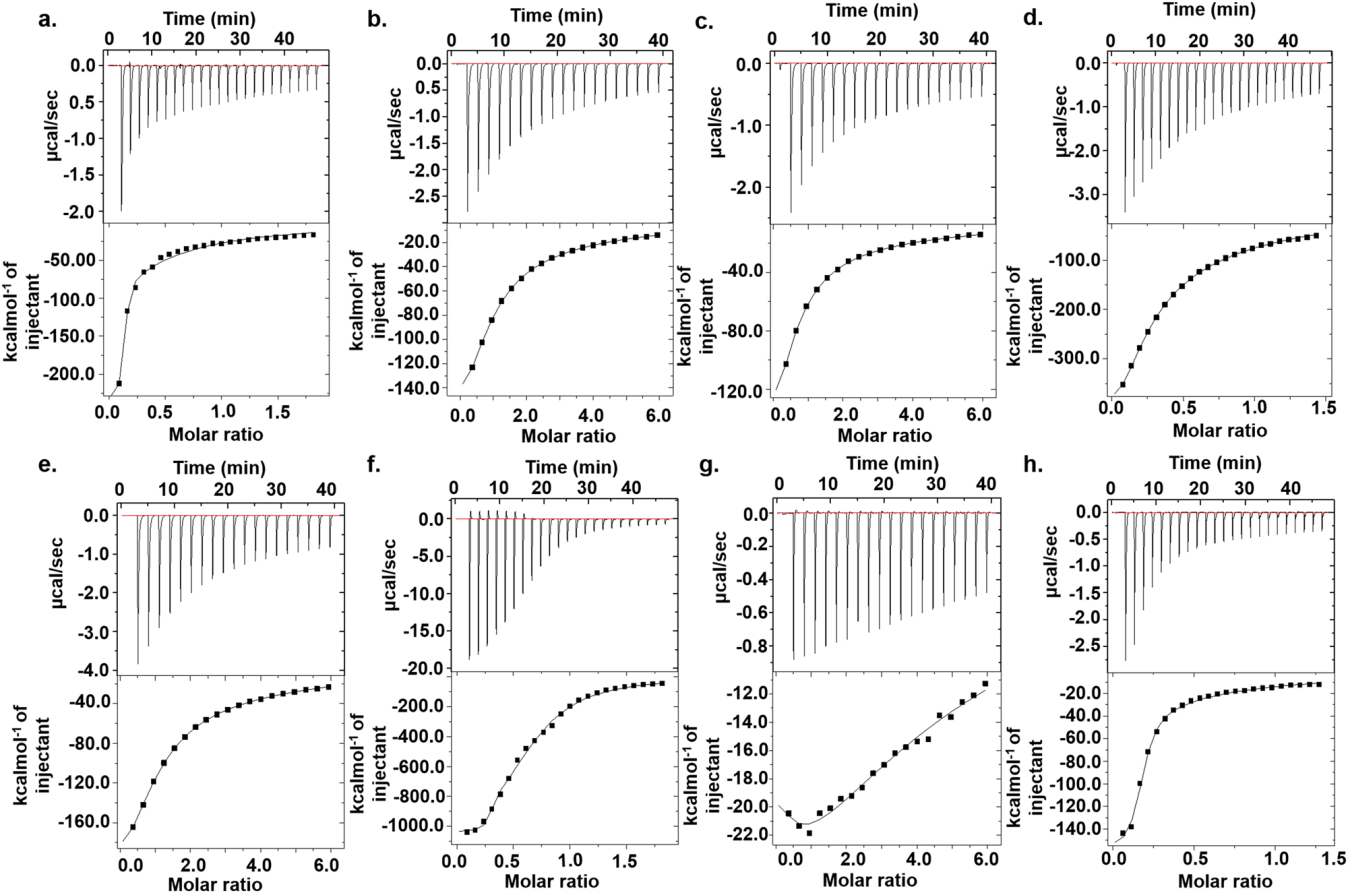
Isothermal titration calorimetry assay of (5′CAG/3′GAC)x6 RNA with compounds (**a**) CP2 (**b**) CP3 (**c**) CP4 (**d**) CP6 (**e**) CP8 (**f**) CP13 (**g**) CP14 (**h**) Myricetin.

Isothermal Titration Calorimetry (ITC) has been considered as a valuable tool to study the interactions of small molecules with different macromolecules including proteins^38^, DNA^39^, and RNA^40^. ITC can detect the heat associated with the chemical reactions between small molecules and macromolecules without the use of the labeled fluorescence or any other modifications. ITC was performed for the compounds that have a better affinity for (5′CAG/3′GAC)x6 RNA (Fig. 2). Out of these seven compounds and Myricetin, CP2, CP6 & CP13 have shown the affinity in the nanomolar range, and their K_d_ values were comparable with the K_d_ values obtained from fluorescence titration study (Supplementary Table S4). To ascertain the selectivity of CP2, CP6, and CP13, we have also performed ITC of these three compounds with 5′CAG/3′GUC duplex RNA as a control (Supplementary Fig. 2). All three compounds have shown K_d_ values in micromolar (µM) range with 5′CAG/3′GUC RNA, indicating their high selectivity for the 5′CAG/3′GAC RNA (Supplementary Table S4). Several studies have stated the fact that the thermodynamic properties, including high association constants, are strongly associated with the high affinity of small molecules with the target^41^. Also, the negative sign in enthalpy changes (∆*H*) stands for the thermodynamically favorable interaction between two molecules. Therefore, the high association constant values of compounds CP2, CP6, and CP13 with high negative ∆*H* are also in favor of high negative free energy of reaction and subsequently supporting the spontaneity of the reaction. After performing the successful screening of these compounds, we proceeded for further studies to assess the specificity of these potential lead compounds with 5′CAG/3′GAC RNA.

### CD, UV melting, and gel mobility assays affirm the specificity of compounds

**Figure 3:**
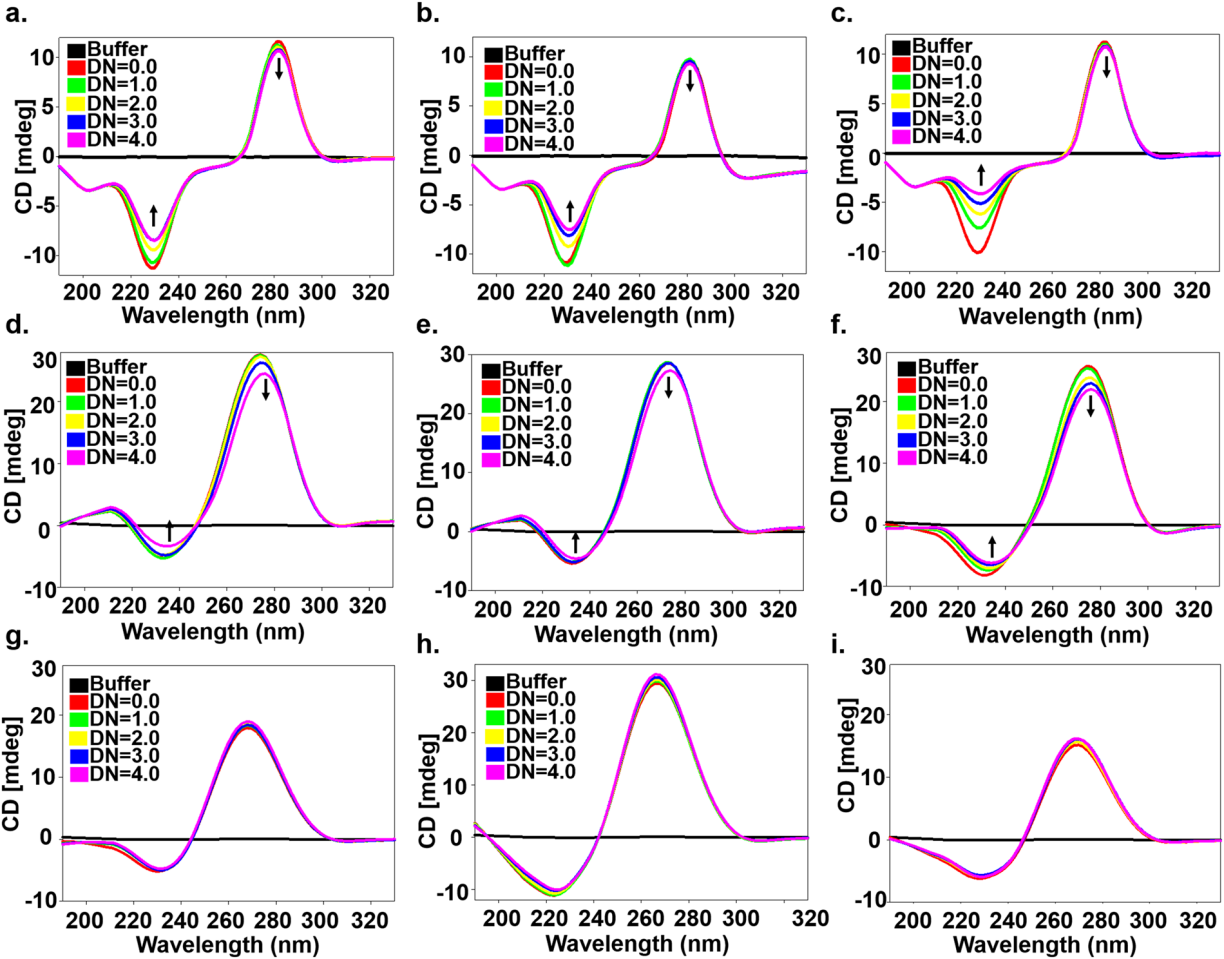
Circular Dichroism spectra of free RNA (20.0 µM) and in the presence of compounds CP2, CP6 & CP13 (**left to right**) with (**a–c**) (5′CAG/3′GAC)x20 (**d–f**) (5′CAG/3′GAC)x6 (**g–i**) (5′CAG/3′GUC)x6.

**Figure 4:**
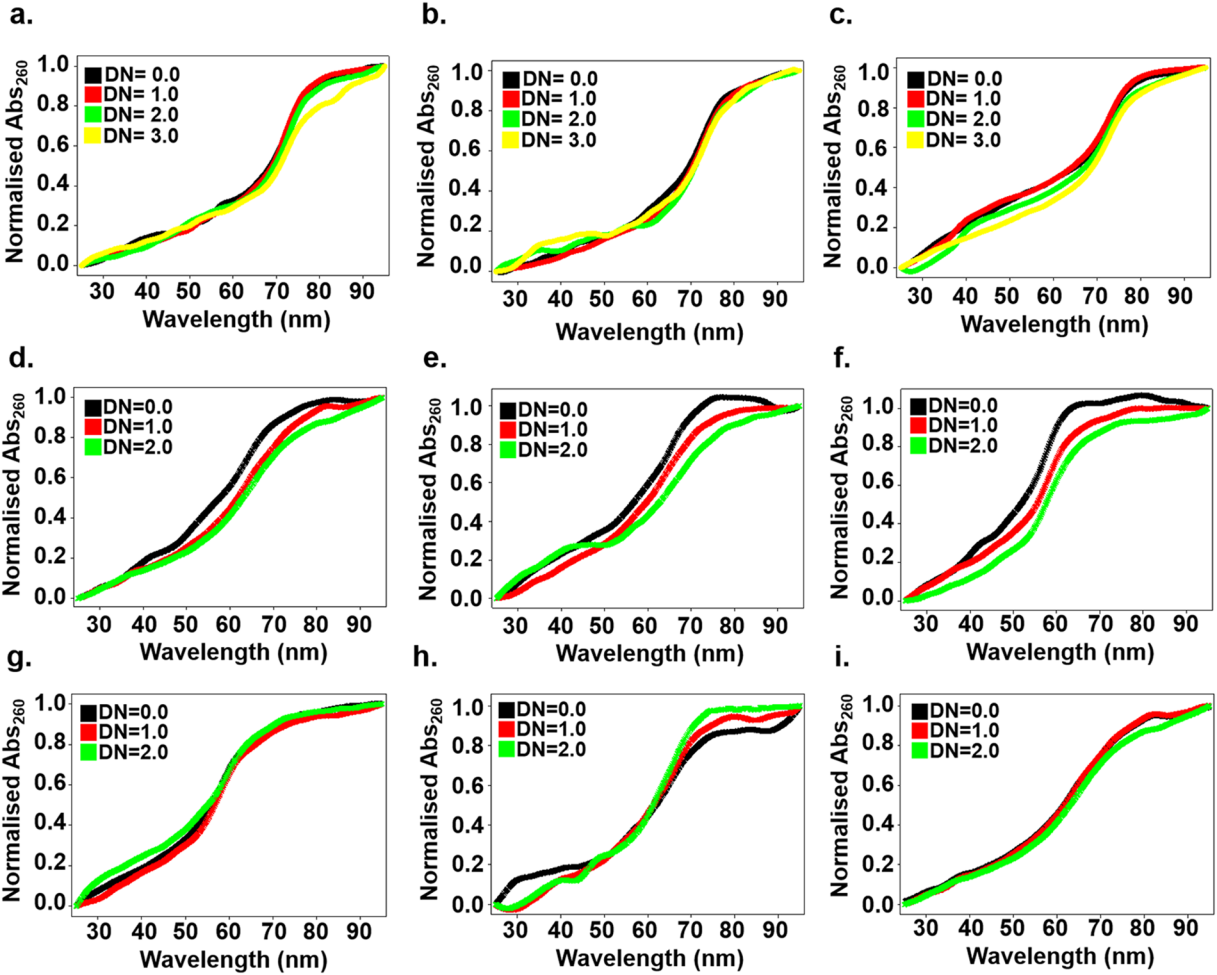
Thermal denaturation curve of free RNA and in the presence of compounds CP2, CP6 & CP13 (**left to right**) with (**a–c**) (5′CAG/3′GAC)x20 (**d–f**) (5′CAG/3′GAC)x6 (**g–i**) (5′CAG/3′GUC)x6.

The binding of small molecules with nucleic acids causes conformational changes; therefore, circular Dichroism has been known as a very valuable technique to assess the binding affinity of compounds with the nucleic acids. Circular Dichroism Spectra of alone RNA was similar to double-stranded A-form-like structure^42^ with a large positive peak near 265–275 and a negative peak near 240 nm. Upon addition of compound in RNA up to DN = 4.0, the positive peak near 270 shifts downward while the negative peak near 240 nm shifted upward in (5′CAG/3′GAC)x6 RNA, which is in accord with the base stacking^43^ of binding compounds with RNA (Fig. 3). Moreover, changes in the CD spectra were prominent in CP6 & CP13 as compared with CP2 however; mere subtle changes were observed for CD spectra of (5′CAG/3′GUC)x6 duplex RNA as a control. Thermal denaturation assay is an advantageous method to deduce the drug binding information^44,45^. Thermal denaturation assays were performed using UV spectrophotometer for CP2, CP6 & CP13 with (5′CAG/3′GAC)x20, (5′CAG/3′GAC)x6 and duplex (5′CAG/3′GUC)x6 RNA (Fig. 4).

Compounds showed up to ∼7–9 °C changes with (5′CAG/3′GAC)x20 and ∼3–5 °C (5′CAG/3′GAC)x6 RNA while no significant change was observed with (5′CAG/3′GUC)x6 RNA (Supplementary Table S5) which were in line with CD spectra results and corroborated the specific binding of compounds with (5′CAG/3′GAC) RNAs. Furthermore, to strengthen our observation, we have proceeded with PCR stop assay and gel retardation assay of the compounds with CAG DNA template and RNA, respectively. PCR stop assay was performed for CP2, CP6, & CP13 with the (5′CAG/3′GAC) and (5′CAG/3′GUC) DNA templates. On addition of the compounds with DNA, the intensity of the band decreased in (5′CAG/3′GAC) DNA however, the reduction in the intensity of (5′CAG/3′GUC) DNA templates were not observed significantly (Supplementary Fig. 3). Encouragingly, the significant reduction in the mobility for (5′CAG/3′GAC) RNA in the presence of compounds whereas, no change in mobility was observed for (5′CAG/3′GUC) RNA. These results were consistent with observed specificity of compounds for (5′CAG/3′GAC) RNA obtained from all the above results. (Supplementary Figure 4).

### Structural insight of compound binding with CAG RNA using NMR spectroscopy and molecular docking

**Figure 5:**
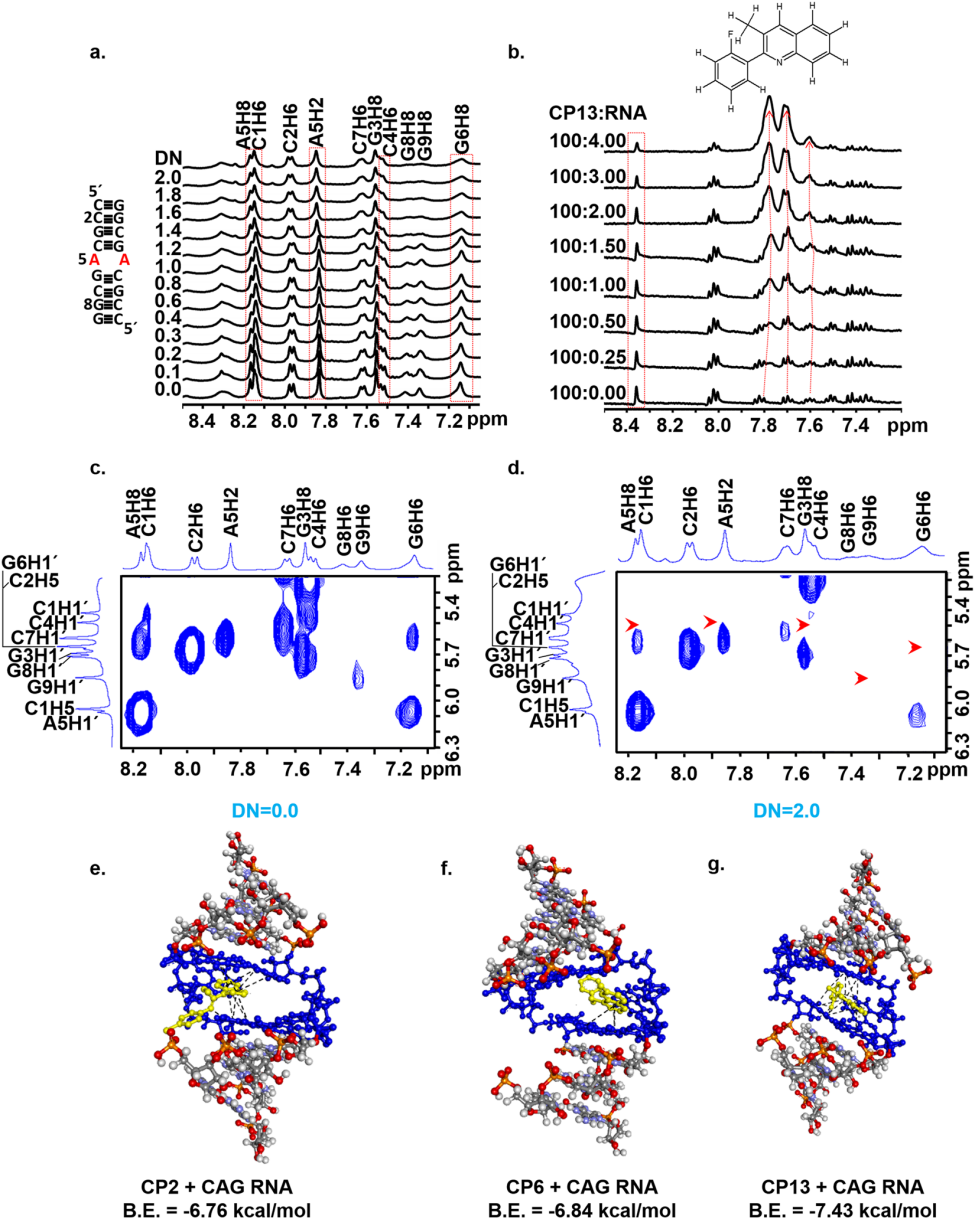
Structural insight into the interaction of 5′r(CCGCAGCGG)3′ with CP13 using NMR spectroscopy. One dimensional proton spectra of RNA as a function of increasing concentration of CP13 showing (**a**) base region (**b**) NMR titration of CP13 with increasing concentration of RNA (**c–d**) Two dimensional NMR spectroscopy of 5′r(CCGCAGCGG)3′ with CP13 at 298 K, at D/N ratios of (**c**) D/N = 0.0 (**d**) D/N = 2.0 (**e–f**) Docked structure of RNA with compounds (**e**) CP2 (**f**) CP6 (**g**) CP13

RNA is a well-formed tertiary structure with deep pockets and cleft surrounded by hydrogen bonding groups. This well-formed tertiary structure provides a legitimate chemical space for small molecules to bind with RNA. The non-canonical A-A base pairing in CAG RNA delivers the conformational flexibility and dynamic behavior, and thus CAG repeats RNA is a suitable target for small molecule-based therapeutics^20,23,46,47^. We have performed the one dimensional NMR spectroscopy to study the binding of our lead compound CP13 with CAG RNA. A short sequence with 1 × AA loop (5′-rC_1_rC_2_rG_3_rC_4_rA_5_rG_6_rC_7_rG_8_rG_9_)-3′ was used, and the proton assignment was done from our previous study^23^. On gradual addition of CP13 to RNA, the change in the shape of proton resonances, as well as the chemical shift, have been observed in RNA (Fig. 5). Interestingly, the chemical shift and changes in proton resonances have been observed either in C_4_A_5_G_6_ triad or involving the terminal protons, and these results were in line with our previous studies^23^. In NMR spectra, the peaks A5H8, A5H2, C4H6, and G6H8 broadened on the addition of CP13 and diminished significantly at DN = 2.0 (Fig. 5a). Moreover, the C4H6 protons get broadened and separate as an individual peak on increasing the concentration of CP13 to RNA.

All these chemical changes observed in C_4_A_5_G_6_ triad also affirm the notion of the specific binding affinity of CP13 with CAG RNA. Furthermore, to assess the involvement of CP13 protons with RNA, we also perform the NMR titration study of CP13 with the gradual addition of CAG RNA. On addition of RNA to CP13 compound, the successive changes in CP13 protons have been observed (Fig. 5b) which also corroborates the binding of CP13 with CAG RNA. Additionally, we have also performed two-dimensional NMR spectroscopy of CAG RNA along with CP13 at different mixing time (Fig. 5). In NOESY spectra, at DN = 2.0, some of the peaks, including C4H6, G6H6, and G9H6, were missing (Fig. 5c,d). G9H6 is terminal peak which could provide an additional binding site for CP13 however, such terminal sequence of C_1_C_2_G_3_ is not feasible in actual pathogenesis condition. In NOESY spectra, some of the peaks got reduced. However, we could not find the cross peaks of CP13 with RNA, which could be due to the overlapping of CP13 resonances with RNA. The absence of NOEs between CP13 and RNA hampered to elucidate the solution structure of CP13-CAG RNA complex. Thus, we intended to perform the molecular docking study with CAG RNA and compounds as we could not be able to elucidate the solution structure due to resonances overlap.

We have conducted the molecular docking studies using Autodock 4.0^48^ (The Scripps Research Institute, CA, U.S.A.). RNA was lined entirely within the grid box, and each of the compounds was blindly docked individually using a Lamarckian Genetic Algorithm (LGA) with defined parameters. Although the compounds were free to bind with any of the bases of the RNA however, compounds preferred to bind with the C_4_A_5_G_6_ triad with lowest binding energy. The most potent conformer of CP2, CP6, and CP13 compounds bind with RNA with the binding energy of −6.76, −6.84 and −7.43 kcal/mole, respectively (Fig. 5e–g). CP13 possess a quinoline moiety which helps to form π-π stacking interaction with the base pairs of RNA. Moreover, the ring attached with the single bond is freely rotatable, which may further enhance the binding with other residues of RNA in its most suitable conformation. The fluorine atom present in the third ring of CP13 also forms strong ligand-receptor interactions due to the non-covalent bonding between the benzene ring of RNA base pairs and halogen atom of the ligand^49^. Moreover, the flexibility of the third ring could provide sites to add some other reactive groups to improve the potency of the compounds further. Several quinoline derivatives are widely reported as neuroprotective compounds due to it’s metal chelating or anti-oxidant activities^50^. NMR spectroscopy and molecular docking studies have substantiated the binding of the compounds with C_4_A_5_G_6_ triad. However, the validation of reducing the CAG RNA and poly Q mediated toxicity is prerequisite for the therapeutic approaches, and therefore we have further validated our compounds in HD and SCA cellular models to assess the efficacy of these compounds.

### Compounds alleviated the poly Q mediated toxicity in HD cellular models

**Figure 6:**
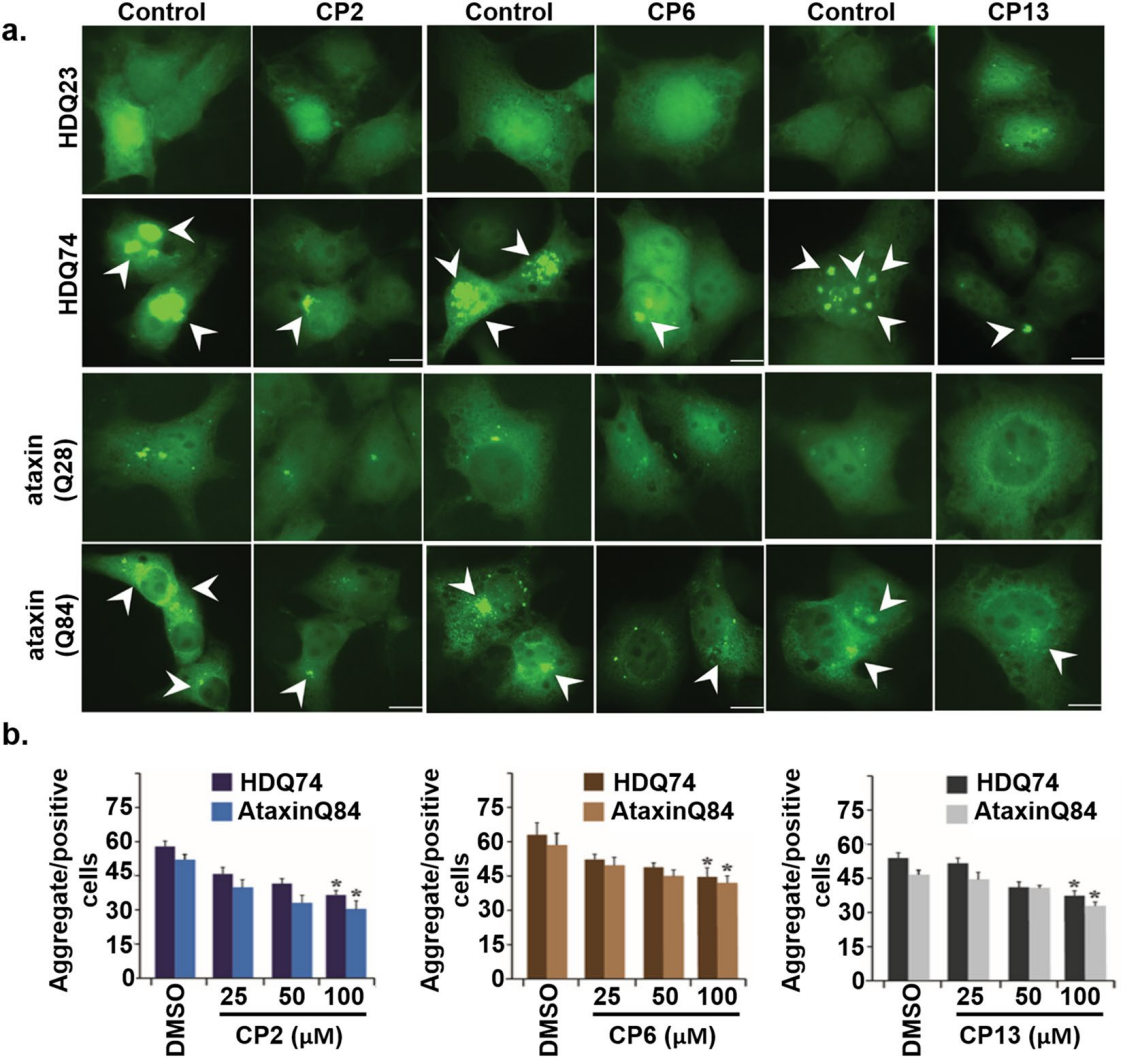
Effect of compounds on expanded polyglutamine aggregates. (**a**) Micrographs of EGFP-HD23Q, EGFP-74Q transiently transfected COS-7 cells, treated with control as well as compounds (100.0 µM) for 12 hours. The arrowheads represent aggregates. (**b**) Cells were transiently transfected with HDQ74 and AtaxinQ84 constructs; after transfection cells were treated with different compounds in a concentration-dependent manner, as shown in the figure. Aggregate positive cells with or without compounds were observed under a fluorescence microscope, and quantification of aggregates was performed with the help micrographs. * p < 0.05 as compared with the control group.

Protein aggregates are the primary hallmark of neurodegenerative diseases. Polyglutamine disorders are a type of neurodegenerative disorders, which are characterized by an abnormal expansion of trinucleotide CAG repeat, which codes for glutamine amino acid^51^. Characterization of drugs that can eliminate these toxic proteinaceous inclusions will be a big help in finding a suitable cure against various protein conformational disorders^52^. Several molecules in the past are identified for their efficacy to remove these toxic proteinaceous aggregates from the cell^53,54^. Previous works have shown the role of protein degradation machinery like autophagy and proteasome in modulating protein aggregation and identifying compounds with the potential of inducing their expression may reduce the toxicity of proteinaceous inclusions^55,56^. We wished to assess the proficiency of the compounds to inhibit the poly Q aggregation. Expanded polyglutamine expressing COS-7 cells were treated in a separate set of experiments with control and compounds (CP2, CP6, and CP13) for 12 hours each. The same sets of cells were then subjected to microscopic fluorescence analysis in independent experiments to examine the formation of aggregate (Fig. 6). Compounds inhibited the poly Q mediated aggregation in higher repeats, Q74, and Q84 repeats whereas no detectable changes have been observed in Q23 and Q28 transfected cells.

**Figure 7:**
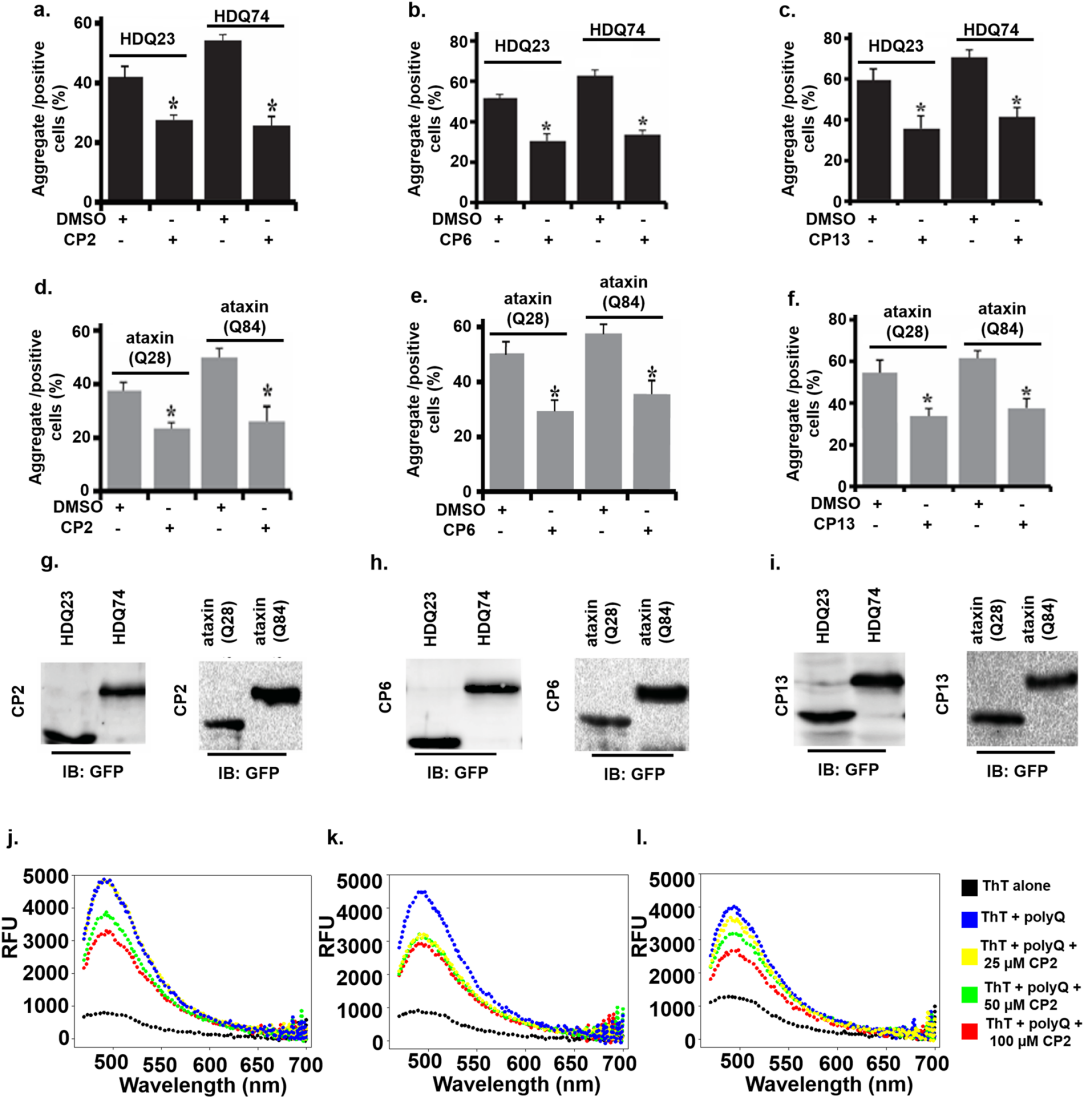
Effect of compounds on polyQ mediated aggregation reduction and Thioflavin assay. (**a–f**) Few same sets of cells expressing expanded polyglutamine proteins were used for quantitative fluorescence analysis of the aggregates. (**g–i**) Plasmid expression analysis of the cells was confirmed by immunoblot, using anti-GFP antibodies (For full blot image, see Supplementary Figure S6b-g) (**j–l**) polyQ aggregation inhibition assay in HD patient-derived cells. Total protein was extracted from HD cells (GM04281) in the absence and presence of compounds and emission spectra of Thioflavin T was recorded. (**j**) CP2 (**k**) CP6 (**l**) CP13

Additionally, to assess the selectivity of the compounds, COS-7 cells were also transfected with the EGFP-CGGx99 plasmid, which has GG mismatch. Interestingly, the reduction in aggregates was observed to some extent in compounds treated cells. However, the reduction in aggregates was not as much significant as in case of (5′CAG/GAC3′) RNA which further corroborates the selectivity of these compounds for (5′CAG/GAC3′) RNA (Supplementary Fig. 6a). Quantitative analysis of aggregate formation for the above set of cells indicates a reduction in expanded polyglutamine aggregate after treatment with all three compounds (Fig. 7).

**Figure 8:**
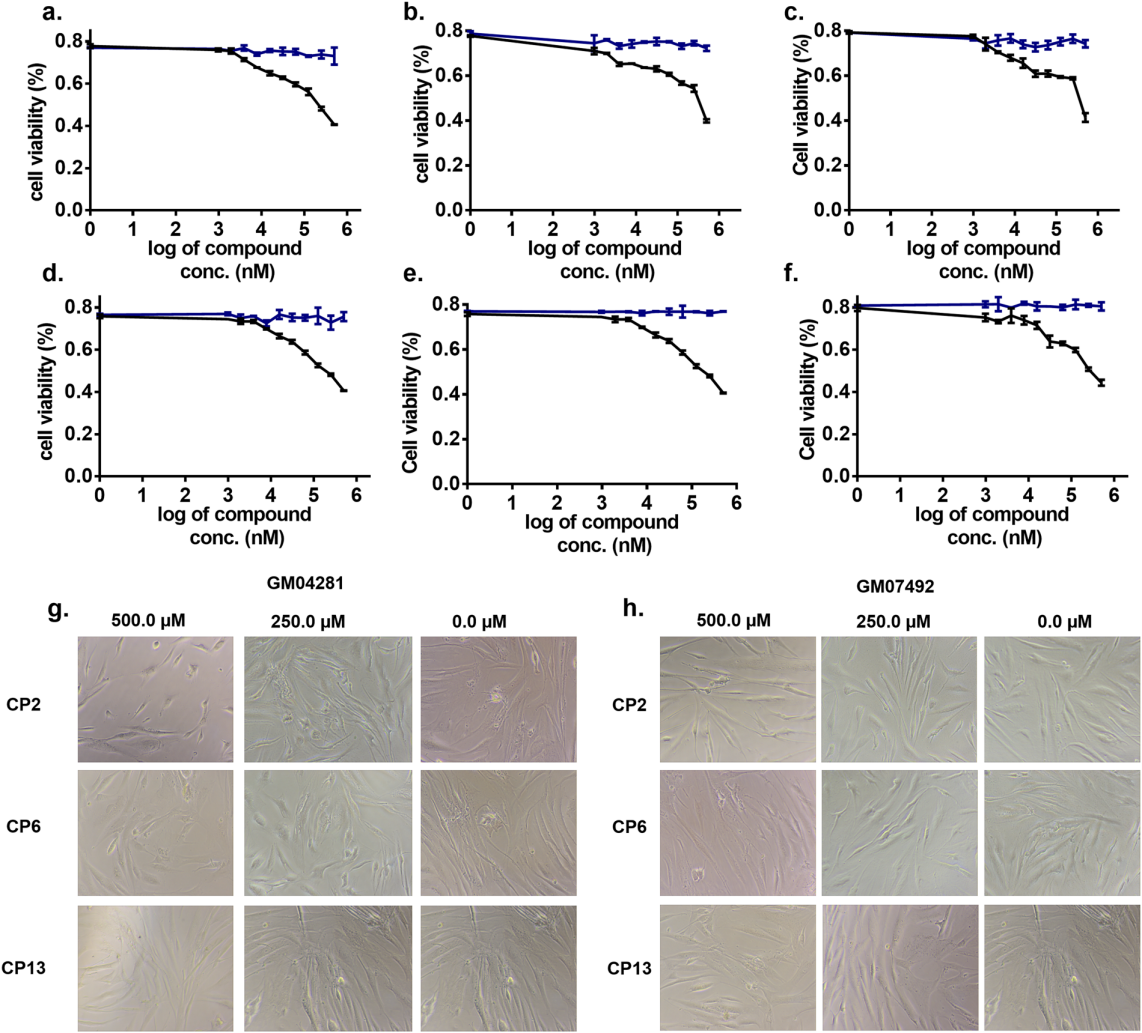
Cell viability assay in HD patient-derived cells treated with compounds (**a–c**) GM04281 cells expressing 74 CAG repeats treated with (**a**) CP2 (**b**) CP6 (**c**) CP13 (**d–f**) GM07492 cells expressing 17 CAG repeats (**d**) CP2 (**e**) CP6 (**f**) CP13 (**g–h**) Bright field micrograph of patient-derived cells treated with compounds (**g**) HD Cells (GM04281) (**h**) Normal fibroblast cells (GM07492). Blue line showing the curve for vehicle (control) while black lines represents cell viability of cells treated with compounds.

Polyglutamine aggregates in cells tend to produce disturbances in cellular mechanisms either by a gain of functions or loss of functions, and that may finally lead to cell death^10,57–59^. As we found that our screened compounds have potential to reduce the size and number of polyglutamine aggregates, therefore, we searched for the role of all three compounds in providing cellular protection against the proteotoxicity of polyglutamine aggregates. The compounds showed very less cytotoxic effects in fibroblast cells from HD patient-derived cells viz. GM04281 and GM07492. GM04281 cells are derived from HD patient, which has approx 74 repeats, while GM07492 cells are derived from a person with approx 17 repeats (Fig. 8, Supplementary Table 6).

Additionally, the cell viability analysis was done by MTT assay for expanded polyglutamine expressing cells exposed to CP2, CP6, and CP13 (Supplementary Fig. 5). An increase in cell viability was observed for all the polyglutamine transfected, and CP2, CP6, and CP13 treated cells as compared to control indicating that all three compounds protect cells from the proteotoxicity of polyglutamine aggregates.

This also strengthens the notion that these compounds could be a promising candidate for reducing the poly Q mediated disease pathogenesis. Furthermore, the validation of expression of normal and expanded polyglutamine plasmids was confirmed by using anti-GFP antibody (Figs. 7g–i) (Full blot images).

In this study, compounds CP2, CP6, and CP13 were tested for their potential in alleviating the cells from proteotoxicity of expanded polyglutamine aggregates of Huntington’s and spinocerebellar ataxias. Different experiments performed during the study, substantiate the effect of the compounds mentioned above in the removal of the cytotoxic proteinaceous inclusions from the cell. A different strategy for evaluating the effect of these drugs on protein aggregation may involve analyzing changes in the level of components of proteins quality control such as E3 ubiquitin ligases like CHIP, MGRN1, ITCH that were previously shown to be effective in modulating accumulation of protein^60–62^. Many drugs in the past have been observed for their effect on protein degradation machinery (like proteasome), e.g., diclofenac and ibuprofen and analysis of the effect of our screened drugs on proteasome or autophagy may provide the molecular mechanism of action of these compounds in alleviating cells from proteotoxicity of protein aggregates^63,64^.

### Effect of compounds in HD patient-derived cells

Thioflavin T (ThT) has been very widely used to probe the protein aggregation in various neurological disorders. ThT has characteristic emission profile with emission maxima at 485 when excited at 440 nm, which significantly increases upon binding with protein aggregates^65^. PolyQ mediated aggregates have been studied using ThT assay previously^66^, therefore, to determine whether studied compounds can alleviate the poly Q mediated aggregation, we have performed the ThT assay for protein extracted from HD patient-derived cells (GM04281) (Fig. 7j–l). In ThT assay, ThT was excited at 440 nm, and emission spectra were recorded at 470–700 nm. The Relative fluorescence units (RFU) were approximately ≤1000, which significantly increased to ~5000 RFU upon addition of protein aggregates.

Moreover, the RFU of cells treated with compounds decreases on increasing the concentration of compounds. All three compounds decrease the RFU of ThT however, the pattern of reducing the RFU was different for all three compounds which could be due to the different mode of binding of the compounds or the mode of action and need to be studied in future (Fig. 7j–l). Lastly, Quinolone based compounds like CP13 not only provides a hope to become therapies for HD and SCAs but also opens the way to target neurological diseases caused by various other trinucleotide repeats RNAs such as CGG, etc. However, further studies involving other action of mechanisms and testing in animal models could provide hope for these compounds to provide therapeutics for these neurological disorders.

### Summary and conclusion

RNA has always been studied vastly, but its role as a therapeutic target was eclipsed for many years. However, recently, a tidal wave of indispensable discoveries towards RNA as a therapeutic target has provided novel avenues in medicine. Trinucleotide repeat RNAs like (CAG)^exp^ have been implicated in various trinucleotide repeat expansion diseases (TREDs) including HD and SCAs. Although the causative agents of these TREDs have been studied many years back, the alleviation of the pathogenesis is a key obstacle. Therefore, therapeutics that could reverse or slow the progression of the pathogenesis of the TREDs is a crucial medical need. The emergence of small molecule-based therapeutics for these TREDs have shown promising avenues. Herein, we have used the shape-based similarity approach for our previously reported compound Myricetin  to delve other potent compounds with high affinity and specificity for (CAG)^exp^ RNA.

Moreover, the biophysical studies, including ITC, CD, UV melting, and NMR spectroscopy studies suggested that the compounds were a potent and selective binder of (CAG)^exp^ RNA. In addition to these preliminary studies, our lead compounds also exhibited the potential to alleviate the polyQ mediated pathogenesis in HD cellular models and cells derived from HD patients. Conclusively, our lead compounds could provide new hope as therapeutics for incurable diseases like HD and SCAs. However, further research involving the animal models and elucidation of the mechanism of action of these compounds is still needed.

## Material and Methods

### Reagents

All Chemicals and solvents like NaH_2_PO4, Na_2_HPO4, (HPLC Grade), D_2_O, DMSO, Sodium Chloride and other cell culture reagents such as MTT were obtained from Sigma-Aldrich Chemicals Ltd. (St. Louis, MO, USA). Tissue culture plates and consumables were purchased from Tarsons Pvt. Ltd. (India) and Thermo Fisher Scientific Inc., (USA). The synthetic RNA oligo (5′-rCrCrGrCrArGrCrGrG-3′) was procured from Integrated DNA technology (IDT) Inc. Different plasmids mentioned below were purchased from Addgene (Cambridge, MA, USA). Lipofectamine 3000, OptiMEM, and Antifade Reagent with DAPI were procured from Life Technologies (USA). Anti-GFP and anti-β-actin antibodies were procured from Santa Cruz Biotechnology, Inc., (USA).

**Plasmids:** pEGFP-C1-Ataxin3Q28 (Addgene-22122), pEGFP-C1-Ataxin3Q84 (Addgene-22123)

### RNA preparation and purification

RNA sequences used for biophysical studies and gel retardation assay were prepared by runoff transcription method using synthetic DNA template as previously reported^36^. Briefly, synthetic DNA templates either amplified by PCR or cloned in a plasmid, were transcribed using T7 RNA polymerase, and transcribed products were purified by denaturing 15% PAGE. After UV shadowing, RNAs were extracted using 0.3 M NaCl by tumbling down for overnight at 4 °C.

### Chemical similarity screening

Virtual screening was done as previously reported^67^. Briefly, virtual screening was done using OMEGA (version 2.5) and ROCS (version 3.2.0.4) software (OpenEye Scientific Software). Discovery studio 4.0 was used to energy minimized (MMFF94 force field). 1000 compounds based on TanimotoCombo scores were ranked as potential hits.

### Fluorescence titration experiments

Fluorescence titration experiments were performed with multi-mode plate reader as previously described^36^. 10.0 μM of RNAs were serially diluted with the last well taken as a blank (No RNA). Each sample was tested in duplicates at RT with a 5′CAG/3′GUC pair served as control. The data was analyzed as per the following equation using SigmaPlot 12.0 software (Systat Software Inc., San Jose California USA), which states for two receptor binding sites with two different affinities k_d_^1^ and k_d_:^2^

1 $${\rm{f}}=\frac{{\rm{B}}ma{x}^{1}\times {\rm{abs}}({\rm{x}})}{{{\rm{k}}}_{{\rm{d}}}1\times {\rm{abs}}({\rm{x}})}+\frac{{\rm{B}}ma{x}^{2}\times {\rm{abs}}({\rm{x}})}{{{\rm{k}}}_{{\rm{d}}}2\times {\rm{abs}}({\rm{x}})}$$

B_max_ = maximum number of binding sites.

K_d_ = equilibrium binding constant.

### Isothermal titration calorimetry (ITC) study

MicroCal iTC200 (GE Healthcare, Biosciences Ltd., Sweden) was used to perform all the ITC studies as previously described^36^. RNAs were prepared in 10 mM Na_2_HPO_4_ buffer (0.1 M NaCl, and 50 mM EDTA, pH = 7.2). RNA was used in the cell, and the compounds were titrated using a syringe with an initial injection of 0.4 μL with 60 s initial delays. The sample was stirred with 750 rpm, and the reference power was set to 8 μcal/s during whole experiment. The two site binding model was used for data fitting to determine the dissociation constant using origin software version 7 (Microcal Software Inc. Northampton, MA, US) provided with the instrument.

### Circular dichroism

Circular Dichroism (CD) experiment was performed on J-815 Spectropolarimeter (Jasco, Inc. Mary’s Court, Easton, Maryland, US) as reported previously^36^. Briefly, RNA (20 μM) was titrated with increasing concentration of compounds in 10 mM Na_2_HPO_4_ buffer (0.1 M NaCl, and 50 mM EDTA, pH 7.2). All the CD studies were performed in a quartz cuvette with a 0.2 cm path length. Spectra were recorded at 0.1 nm intervals from 200 nm to 300 nm with a 1 nm-slit width and averaged over three scans. Finally, data were normalized by subtracting the buffer CD spectra from RNA CD spectra as well as RNA-Drug complex CD spectra.

### Thermal denaturation experiments

Thermal denaturation studies were performed as previsouly published method^36^. using Perkin Elmer Lambda 35 spectrophotometer equipped with a Peltier temperature programmer (PTP 6 + 6) and water Peltier system PCB-1500. The change in absorbance at 260 nm at a rate of 1 °C/minute in the absence and presence of compounds at D/N ratio of 2.0 and D/N ratio of 3.0 was monitored upon heating of RNA to 25 °C to 95 °C. The change in thermal denaturation was calculated by normalizing the changes in absorbance at 260 nm vs. temperature using the SigmaPlot 12.0 software.

### Gel retardation assay

5 µM of each RNA (AA and AU pair) was incubated with decreasing concentration of compounds (1000.0–0.0 µM) and kept for 30 minutes at room temperature. 3% agarose gel stained with ethidium bromide was used to assess the inhibition in movement of bands. Finally, the inhibition of movement in bands was analyzed using ImageQuant LAS 4000 (GE Healthcare, Biosciences Ltd., Sweden).

### PCR Stop assay

PCR stop assay was performed as previously reported by our group^36^. Briefly, the Template for AA × 6 (5′- GGA GAG GGU UUA AUC AGC AGC AGC AGC AGC AGU ACG AAA GUA CAG CAG CAG CAG CAG CAG AUU GGA UCC GCA AGG - 3′) and complementary sequence (5′- GGC CGG ATC CTA ATA CGA CTC ACT ATA GGG AGA GGG TTT AAT - 3′) were procured from Sigma-Aldrich Chemicals Ltd. (St. Louis, MO, USA). The reaction was performed in a master mix consisting 1X PCR buffer, 0.33 mM dNTPs, 4.25 mM MgCl_2_, 2 μM oligonucleotide, 2.5 units Taq DNA polymerase (Sigma-Aldrich Chemicals Ltd. St. Louis, MO, USA) and a dose of titration of ligands. The thermal cycling conditions were as follows: 95 °C for 5 min, followed by 25 cycles of 95 °C for 30 s, 50 °C for 30 s, 72 °C for 1 minute and finally held at 4 °C following completion. The amplified products were mixed with 6X DNA loading dye and resolved on 3% agarose gel stained with ethidium bromide. The gel image was analyzed using ImageQuant LAS 4000 (GE Healthcare, Biosciences Ltd., Sweden).

### Nuclear magnetic resonance spectroscopy

Nuclear Magnetic Resonance (NMR) experiments for RNA duplex with a single CAG motif, 5′-rCrCrGrCrArGrCrGrG-3′ were performed on a high-resolution AVANCE III 400 or 700 MHz BioSpin International AG, Switzerland as reported previously^23^. RNA samples were prepared in an appropriate buffer, and 3 - (Trimethylsilyl) propionic-2, 2, 3, 3-d 4 acid sodium salt (TSP) was used as a reference. H2O + D2O solvent at a 9:1 ratio was used for all titration studies and 64 K data points were recorded for 1D proton NMR spectra. Topspin (3.5 versions) was used to process, integrate, and analyze the data.

### Docking studies

The molecular docking of RNA and compounds was described earlier^36^. The previously reported structure of CAG duplex RNA (PDB code:2MS5^47^) was utilized as the starting model. The structure was refined further using CHARMM force field performed on Discovery studio3.5 (San Diego, Dassault Systèmes, USA) for the necessary replacements, optimization of structure and ligands structures and the addition of residues. Autodock 4.0^48^ (The Scripps Research Institute, La Jolla, CA, U.S.A.) was used for the docking study with RNA as a rigid body. Default values were used for all other paramters. RNA and ligand structures were converted to AD4 format files, and Gesteiger charges were assigned to the atoms. The grid was arranged so that it covers complete RNA structure so that ligand can explore the whole conformational space. The Lamarckian genetic algorithm was used for the search, and the results were analyzed based on binding energy.

### Ethics statement

The following cell lines GM07492 and GM04281 were obtained from the NIGMS Human Genetic Cell Repository at the Coriell Institute for Medical Research. The Coriell Institute maintains the written consent forms and privacy of the donors of the samples, and the authors had no contact or interaction with the donors.

### Cell culture

All the Fibroblasts cells were procured from the Coriell Cell Repositories (Coriell Institute for Medical Research, New Jersey, USA). An unaffected individual (GM07492 line) expresses normal repeat tract, r(CAG)17 while HD patient (GM04281), expresses expanded repeats, r(CAG)69. All the cell lines were grown in appropriate medium supplemented with 10% FBS and 1x antibiotic-antimycotic solution.

### Immunocytochemistry and aggregation counting

The immunocytochemistry and aggregation counting assays were performed as previously reported^36^. Breifly, COS-7 cells were transiently transfected with the desired plasmid. The medium was replaced with fresh medium containing with either DMSO or compounds of interest, after 6–8 hour post-transfection. The cells were washed with PBS and fixed with 4% paraformaldehyde for 15–20 minutes and further permeabilized with 0.5% Triton-X 100 for 5 minutes for immunofluorescence assay. Afterward, 2–3 washing with PBS and mounting with DAPI was done and finally, fluorescence microscope was used to capture the images. Aggregates counting were done by using few of the same set of cells at lower magnification.

### Western blot and MTT assays

Briefly, COS-7 cells were transiently transfected with desired plasmids (EGFP-HDQ23, EGFP-HDQ74, ataxin-3(Q28) and ataxin-3(Q84)). Each set of transfected cells was treated either with DMSO or compounds of interest for 12 hours each. Immunoblot analysis was done with same set of cells^68^. For Cell viability assay, cells viability assay was performed in triplicates via MTT assay as described in a previous publication^69^.

### Thioflavin T fluorescence assay

Thioflavin T assay fluorescence was performed for total protein extracted from HD patient-derived cells treated with compounds of interest. Cells were seeded in 24 well plates for, and media containing a compound of interest was added at 80% confluency of the cells. After 24 hours, the cells were lysed using RIPA lysis buffer and total protein were  isolated. At the day of the experiment, 2.5 mM Thioflavin-T (ThT) stock solution was prepared in phosphate buffer and filtered through 0.22 µm filter (Millipore). ThT dye (10 µM final concentration) was applied to untreated (control) and treated sample. ThT alone, as well as the sample containing ThT dye, was excited at 440 nm and emission spectra were recorded at 470–700 nm. SigmaPlot 12.0 software (Systat Software Inc., San Jose California USA) was used to plot the emission spectra of each sample.

## Supplementary information

Supplementary Information
