# Supplementary material for: Discovery of a potent small molecule inhibiting Huntington’s disease (HD) pathogenesis via targeting CAG repeats RNA and Poly Q protein
Source: Sci Rep. 2019 Nov 14;9:16872. doi: 10.1038/s41598-019-53410-z (PMC6856162; doi:10.1038/s41598-019-53410-z)
Supplement: Supplementary file 1 — Supplementary Information [file 41598_2019_53410_MOESM1_ESM.pdf]

Discovery of a potent small molecule inhibiting Huntington's disease pathogenesis *via* targeting CAG repeat RNA and Poly Q protein

Eshan Khan[a], Subodh Kumar Mishra[a], Ribhav Mishra[b], Amit Mishra[b], and Amit Kumar[a†]

[a] Discipline of Biosciences and Biomedical Engineering, Indian Institute of Technology Indore, Simrol, Indore 453552, India

[b] Cellular and Molecular Neurobiology Unit, Indian Institute of Technology Jodhpur, Rajasthan 342011, India

[†]**Correspondence:** Amit Kumar, Associate Professor, Discipline of Biosciences and Biomedical Engineering, Indian Institute of Technology Indore, Simrol, Indore 453552, India Tel: +91-731-2438-771; Fax: +91-731-2438-721; Email: amitk@iiti.ac.in; amitkdbs@gmail.com

Table of Contents		
**Supplementary Tables**		**S10-S17**
**Supplementary Table 1**	Chemical structures of the small-molecules used in screening	**S10-S12**
**Supplementary Table 2**	The binding constant values of compounds with one motif RNA	**S13**
**Supplementary Table 3**	The binding constant values of compounds with multiple motif RNA	**S14**
**Supplementary Table 4**	The binding constant values of compounds with multiple motif RNA using ITC	**S15**
**Supplementary Table 5**	The melting temperature values of different 5'CAG/3'GAC & 5'CAG/3'GUC RNA motif in absence and presence of compounds	**S16**
**Supplementary Table 6**	The IC50 values of compounds with HEK293 cells calculated by MTT assay	**S17**
**Supplementary Figures**		**S18-23**
**Supplementary Figure 1**	Fluorescence titration curves of RNAs with compounds	**S18-S19**
**Supplementary Figure 2**	Plots of Isothermal titration calorimetry	**S20**
**Supplementary Figure 3a**	PCR stop assay	**S21**
**Supplementary Figure 3b**	Quantification of PCR stop assay	**S22**
**Supplementary Figure 4a**	Gel retardation assay	**S23**
**Supplementary Figure 4b**	Quantification of Gel retardation assay	**S24**
**Supplementary Figure 5**	Cell viability assay	**S25**
**Supplementary Figure 6**	Western blot analysis full images	**S26**

CP1=NSC351550 M.W.= 256.28

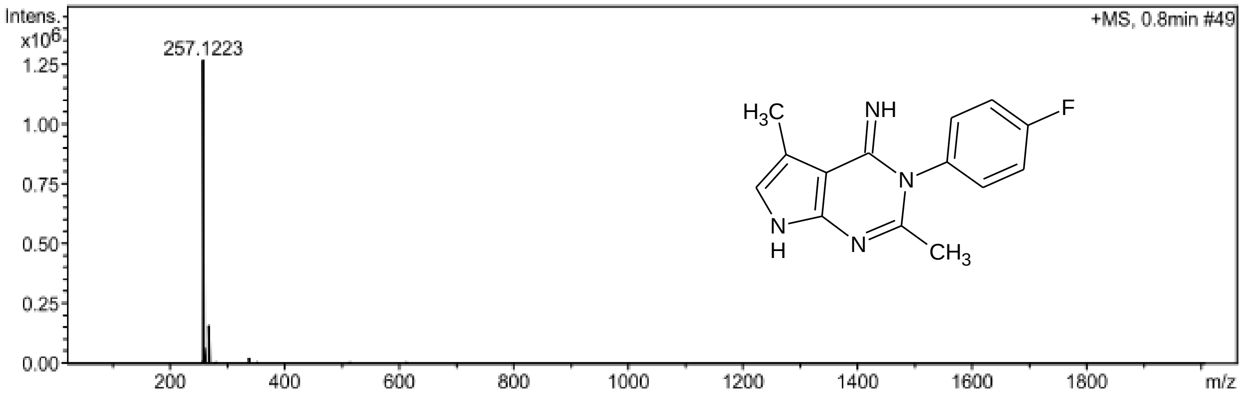

CP2= NSC52101, M.W. = 314.83

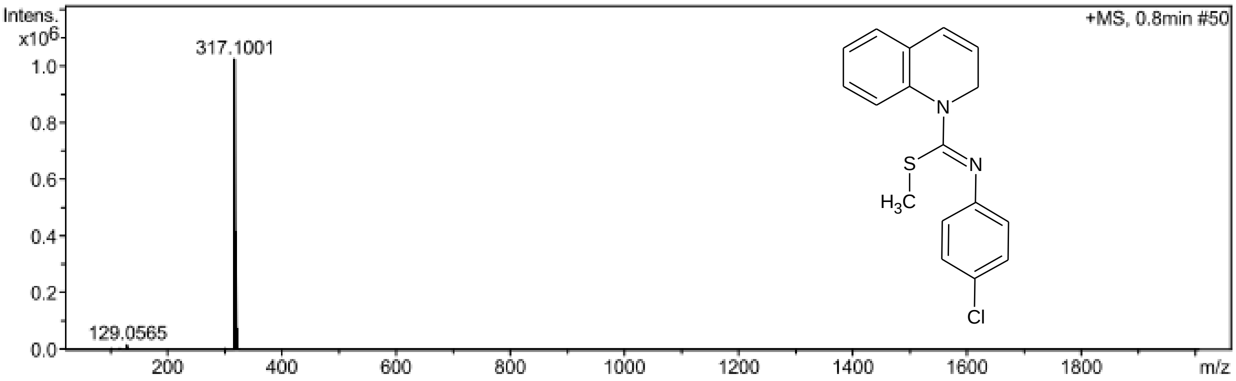

CP3= NSC47923, M.W. = 263.33

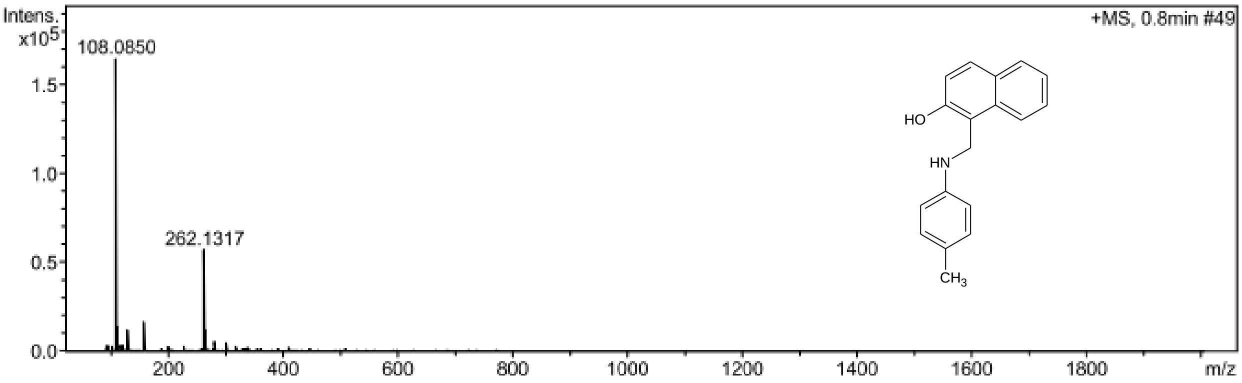

CP4= NSC351310, M.W. = 220.27

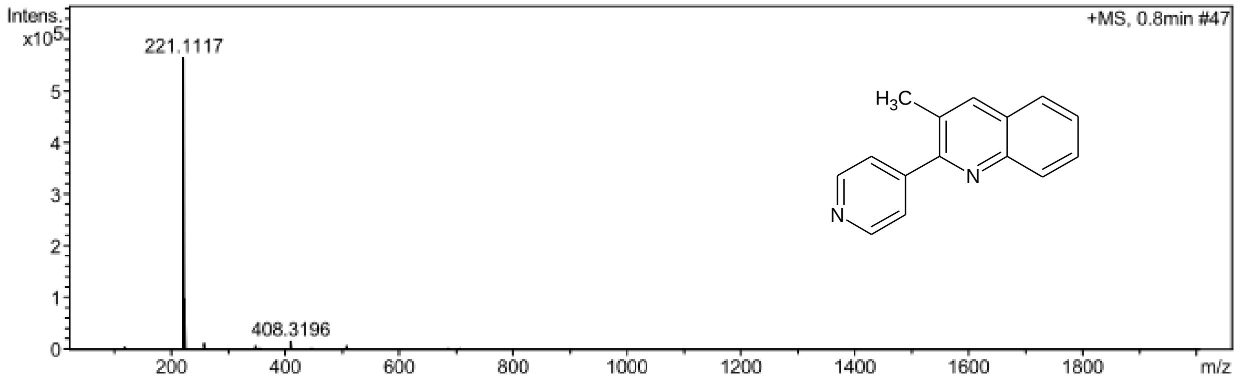

CP5= NSC101815, M.W. = 290.36

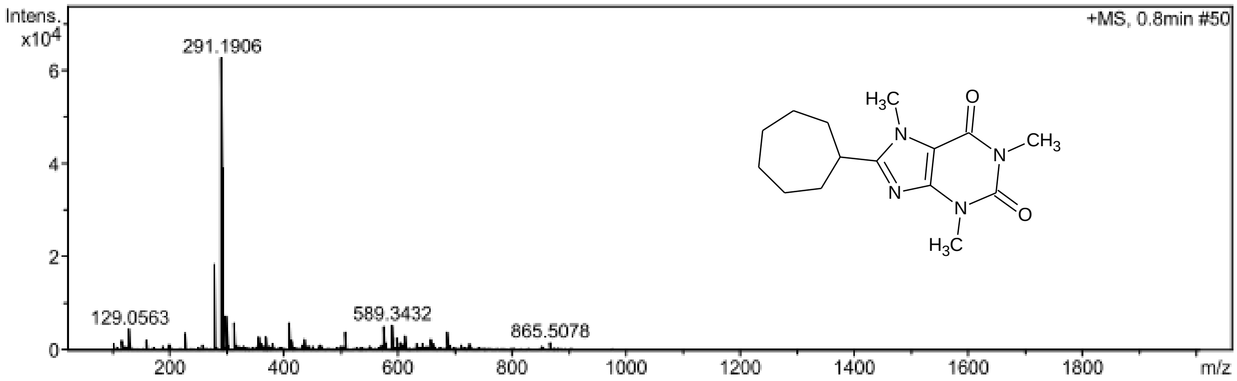

CP6= NSC66098, M.W. = 270.32

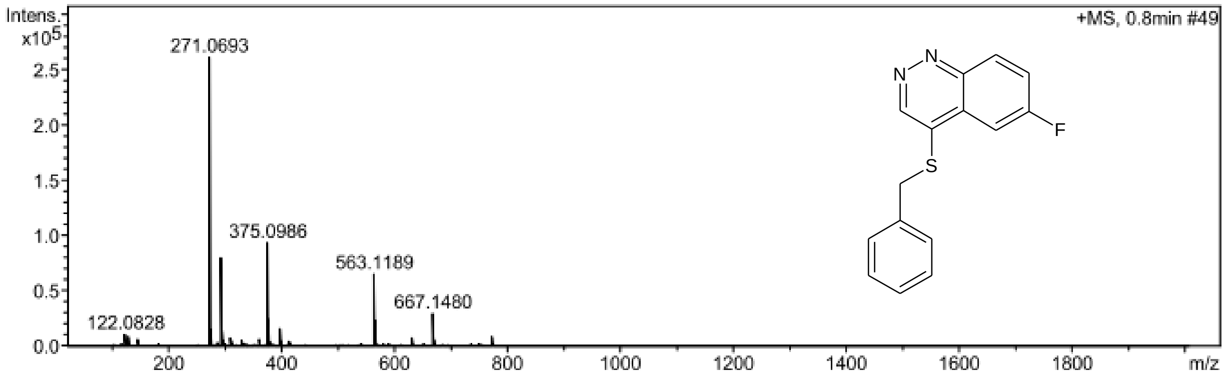

CP7= NSC12769, M.W. = 255.70

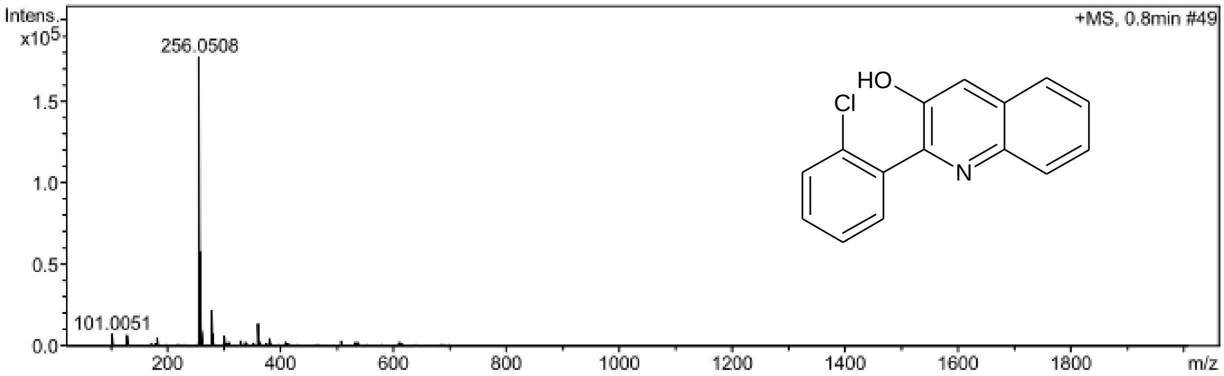

CP8= NSC14378, M.W. = 265.33

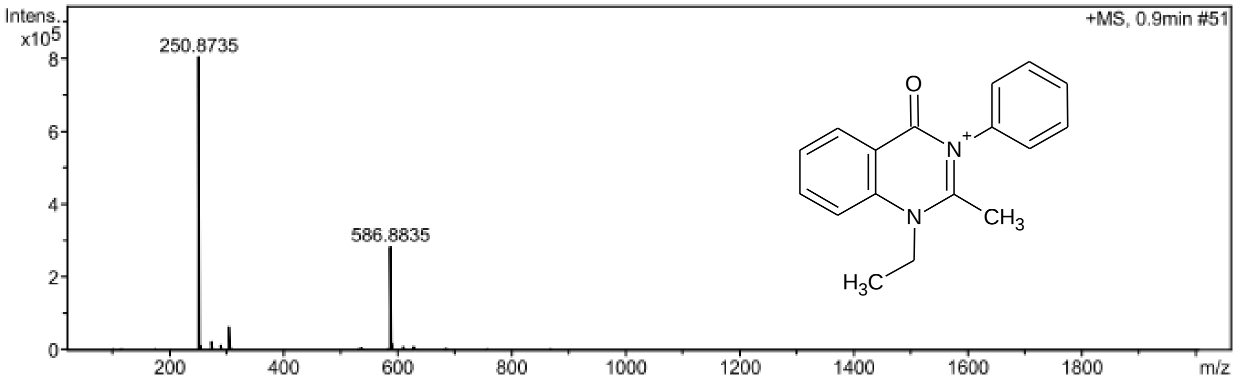

CP9= NSC65028, M.W. = 253.25

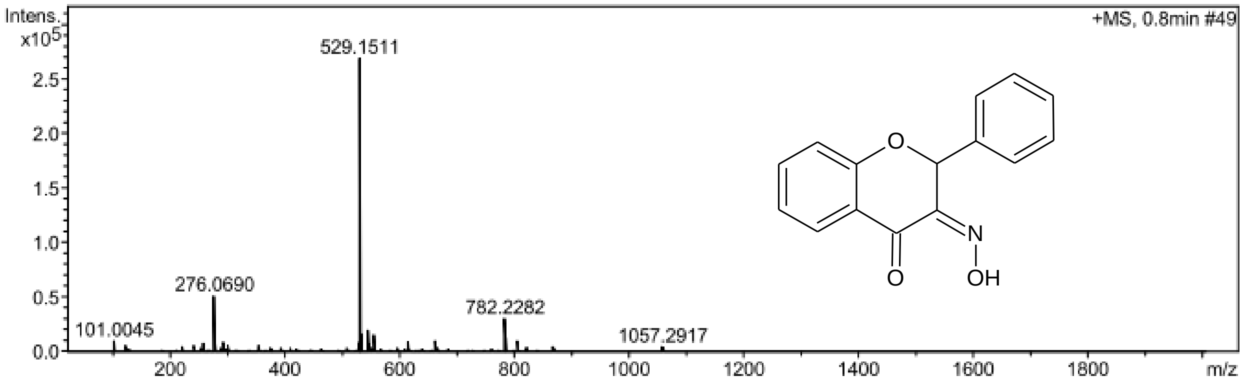

CP10= NSC66104, M.W. = 250.27

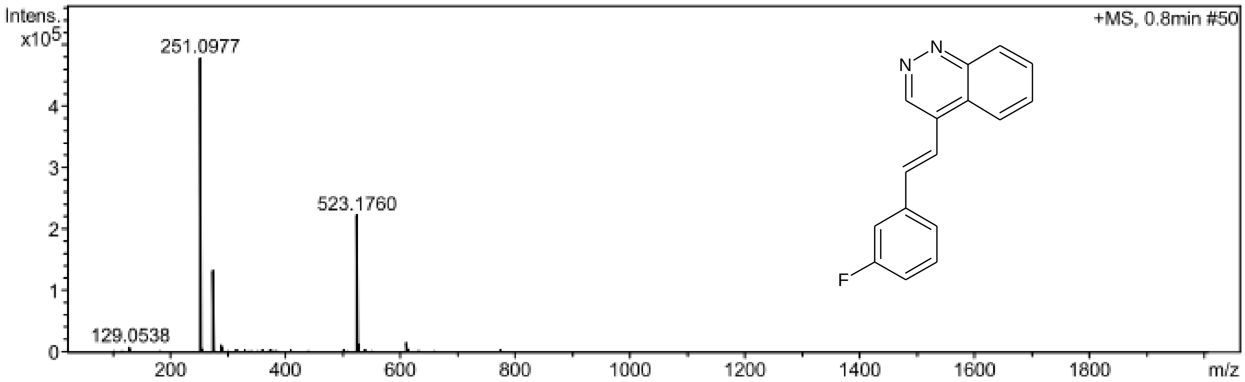

CP11= NSC97487, M.W. = 224.25

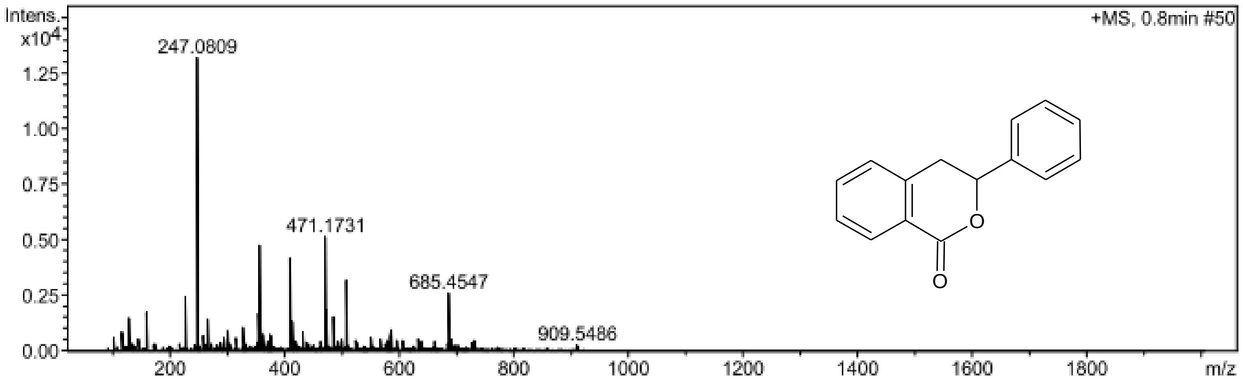

CP12= NSC12823, M.W. = 284.15

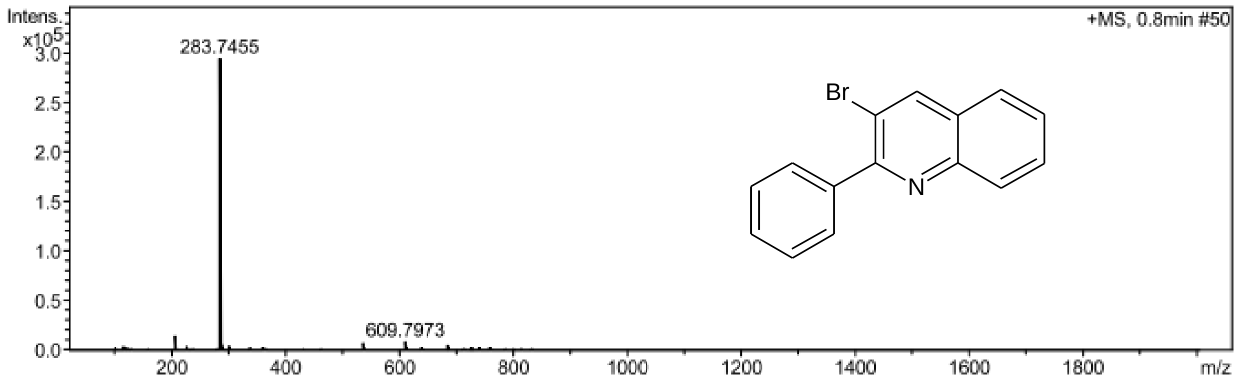

CP13= NSC109779, M.W. = 237.27

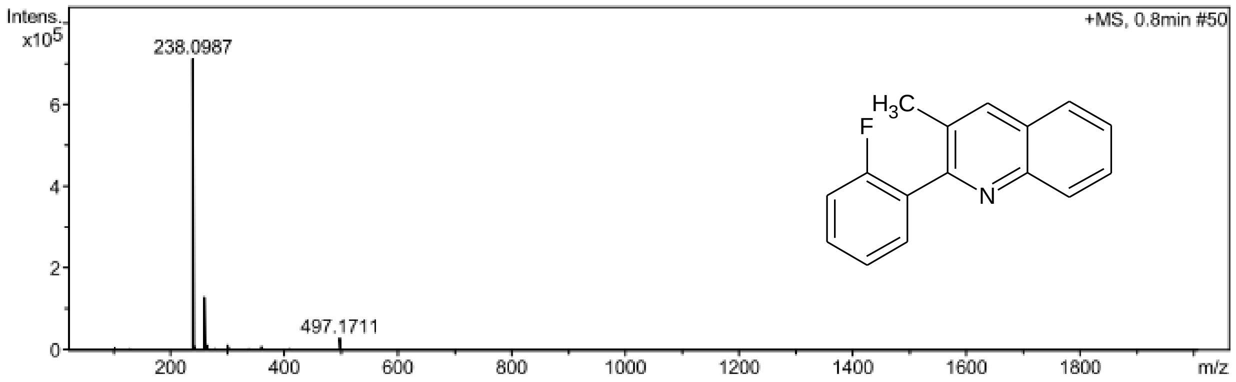

CP14= NSC25678, M.W. = 277.32

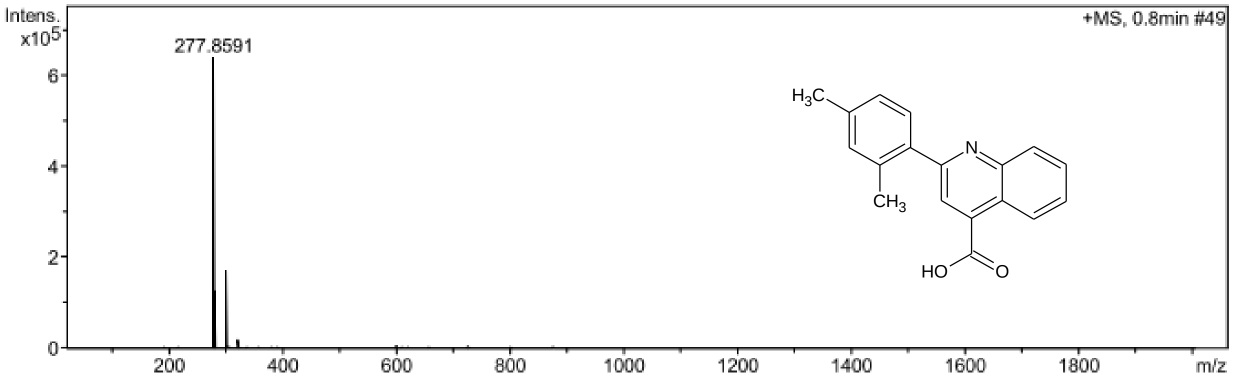

CP15= NSC12776, M.W. = 297.74

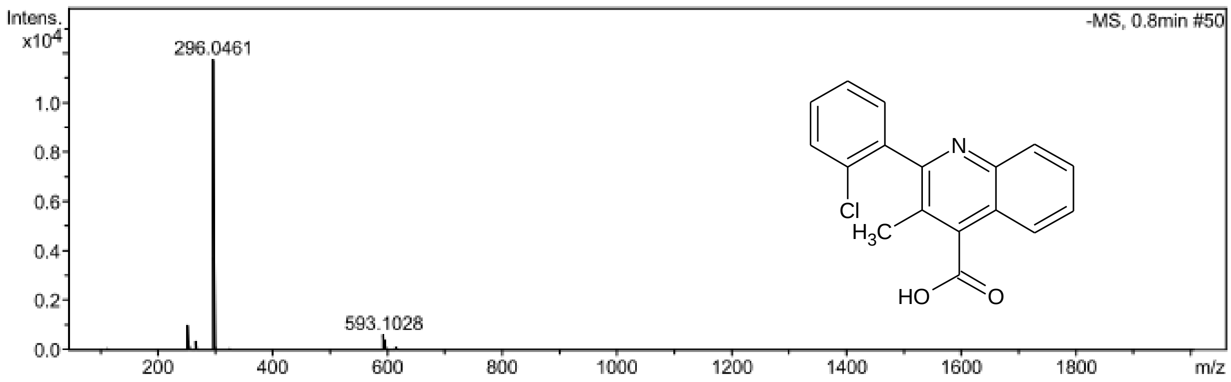

CP16= NSC14049, M.W. = 233.31

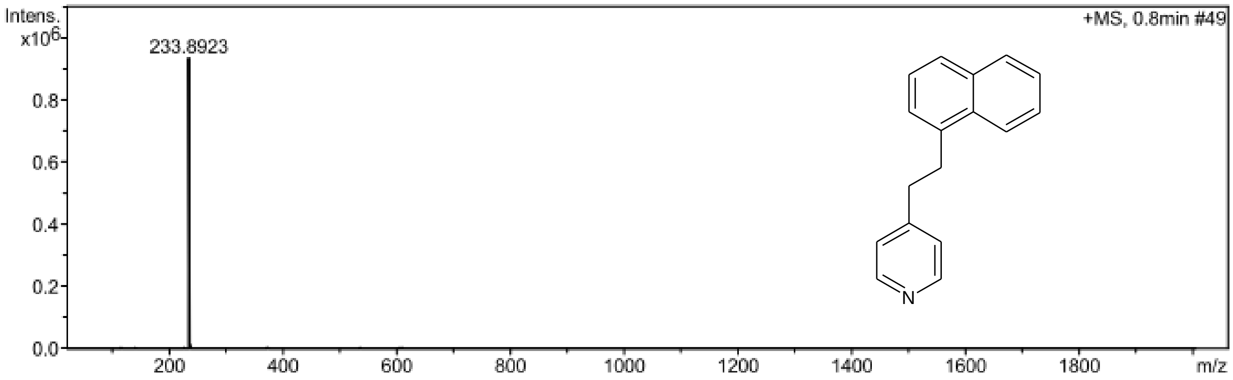

CP17= NSC354463, M.W. = 265.31

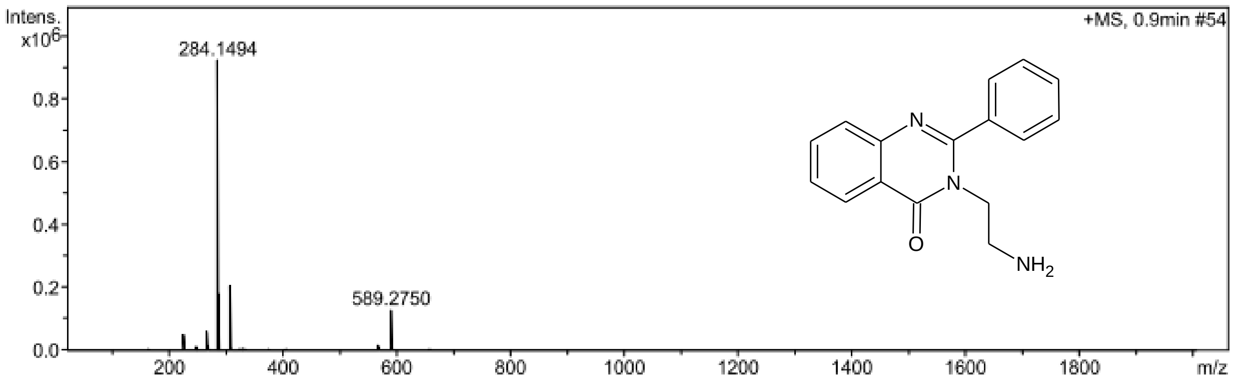

CP18= NSC109780, M.W. = 254.71

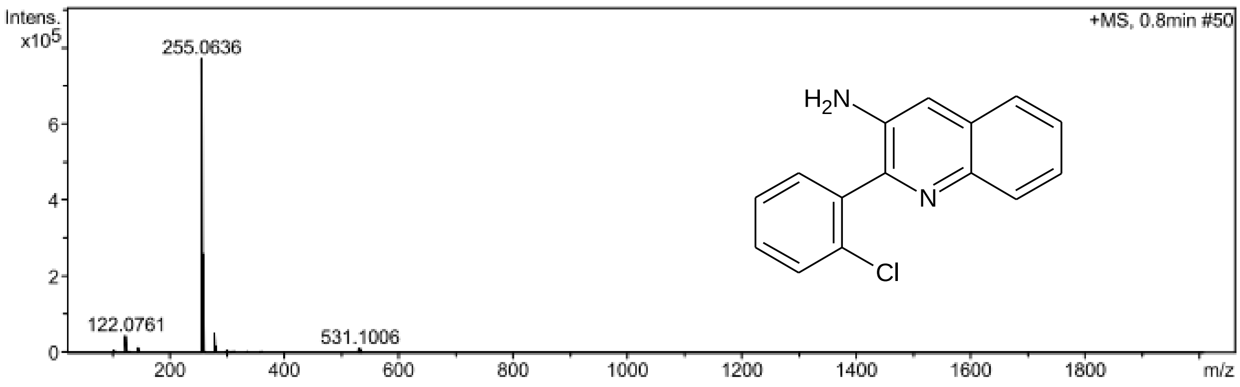

CP19= NSC401369, M.W. = 250.31

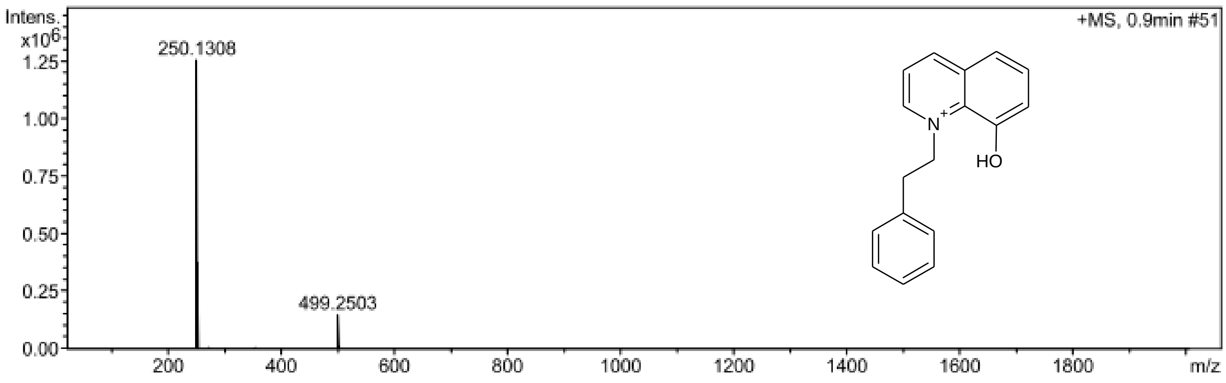

Table S1 Chemical structures of the small-molecules used in screening for repeats containing RNA

Compound Code	Structure
CP1	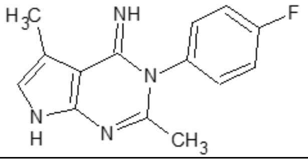
CP2	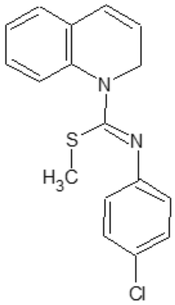
CP3	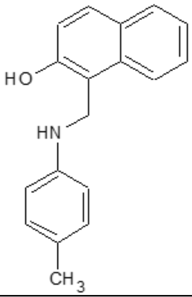
CP4	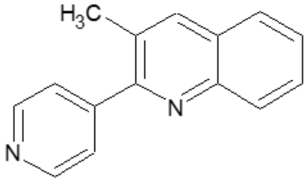
CP5	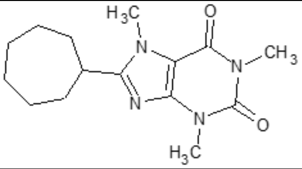
CP6	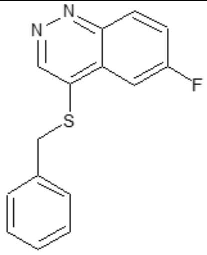
CP7	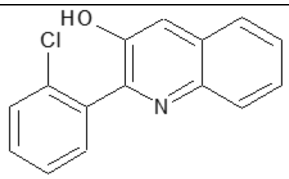

CP8	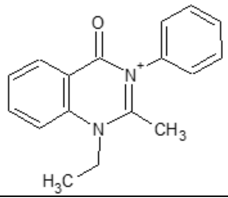
CP9	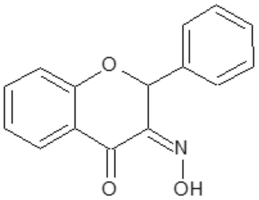
CP10	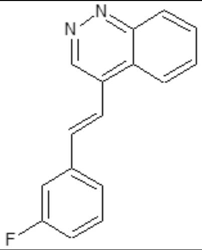
CP11	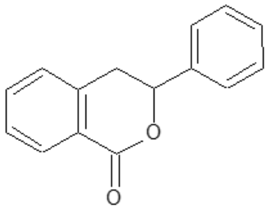
CP12	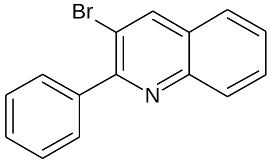
CP13	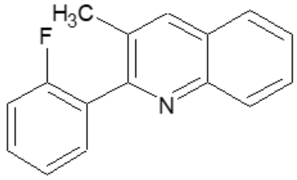
CP14	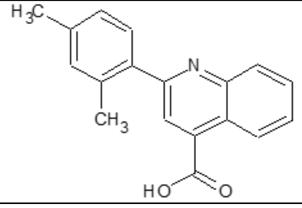
CP15	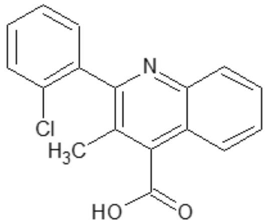

CP16	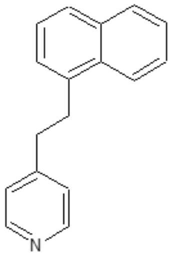
CP17	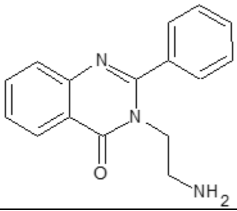
CP18	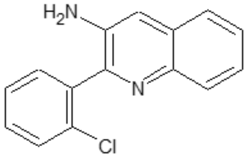
CP19	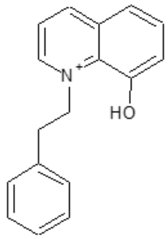

Table S2: The binding constant (K_d (μM)) values of compounds with RNA 5' CAG/3'GAC and 5' CAG/3'GUC

Compound	Binding constant values (μM)			
	RNA 5' CAG/3'GAC		RNA 5' CAG/3'GUC	
	K_d1	K_d2	K_d1	K_d2
**CP1**	0.1672	11.4500	0.0280	0.4521
**CP2**	0.0540	2.1215	0.5662	0.5662
**CP3**	0.0893	1.5579	0.2031	0.7697
**CP4**	0.0009	0.7255	0.0092	0.7178
**CP5**	1.3840	1.3840	0.6657	0.6657
**CP6**	0.0476	0.9693	0.7339	0.7339
**CP7**	0.0178	2.4435	0.0005	1.1453
**CP8**	0.0103	4.7000	0.6154	0.6154
**CP9**	0.6778	$1.91x10^7$	0.0124	0.9227
**CP10**	0.9087	$1.81x10^7$	0.4957	0.4957
**CP11**	1.4307	1.4307	1.2764	1.2764
**CP12**	2.1650	2.1650	0.6115	0.6115
**CP13**	0.3455	1.0217	1.2717	1.2717
**CP14**	0.0675	4.1180	0.5012	1.9855
**CP15**	0.0135	0.1948	0.0172	0.6402
**CP16**	1.5816	1.5816	0.4933	0.4933
**CP17**	N.D.	N.D.	N.D.	N.D.
**CP18**	1.0361	1.0361	0.6931	0.6931
**CP19**	0.3463	4048.3000	N.D.	N.D.

Table S3: The binding constant (K_d, (µM)) values of compounds with multiple loop RNAs

Compounds	Binding constant values (µM)			
	RNA (5'CAG/3'GAC)x6		RNA (5'CAG/3'GUC)x6	
	K_d1	K_d2	K_d1	K_d2
**CP2**	0.0003	0.1749	0.6203	0.6203
**CP3**	0.0495	0.1588	0.2452	0.2452
**CP4**	0.2188	0.49	0.4407	0.7459
**CP6**	0.0005	0.1344	0.2021	0.2021
**CP8**	0.2061	0.1768	0.1150	0.1758
**CP13**	0.0015	0.387	0.3038	0.3038
**CP14**	0.1823	0.1823	0.2823	0.2823
**Myricetin**	0.0730	0.4699	N.D.	2.4757

Table S4. The binding constant (K_d) values of compounds with multiple loop RNAs calculated using ITC experiment.

Compounds	Binding constant values			
	RNA (5'CAG/3'GAC)x6		RNA (5'CAG/3'GUC)x6	
	K_d^1 (μM)	K_d^2 (μM)	K_d^1 (μM)	K_d^2 (μM)
CP2	0.028	12.3	9.52	N.D.
CP3	27.700	2.13	-	-
CP4	62.500	3.18	-	-
CP6	0.709	10.86	30.3	0.42
CP8	38.010	2.35	-	-
CP13	0.011	1.54	3.89	174.2
CP14	60.200	6.89	-	-
Myricetin	0.253	26.24	-	-

Table S6. The IC_{50} values of compounds with HEK293 cells calculated by MTT assay

**Compounds**	**IC_{50} Values (μM)**		
	**HEK293 Cells**	**Normal fibroblast cells**	**HD cells**
**CP2**	19.06	56.33	46.63
**CP3**	42.44	-	-
**CP4**	76.99	-	-
**CP6**	40.24	55.30	44.44
**CP8**	17.38	-	-
**CP13**	62.86	53.17	52.70
**CP14**	17.25	-	-
**Myricetin**	22.45	-	-

Table S5. The melting temperature values of different 5'CAG/3'GAC & 5'CAG/3'GUC RNA loop in absence and presence of compounds

Compounds	Drug/Nucleic acid ratio	Melting temperature (°C) of different loop containing RNAs		
		AAx20	AAx6	AUX6
**CP2**	DN0.0	67.6	57.6	61.8
	DN1.0	70.4	61.8	61.9
	DN2.0	72.1	63.0	62.5
	DN3.0	75.2	-	-
**CP6**	DN0.0	67.8	57.0	60.4
	DN1.0	70.2	59.8	60.8
	DN2.0	71.9	62.8	61.4
	DN3.0	74.9	-	-
**CP13**	DN0.0	66.8	51.8	62.0
	DN1.0	69.1	55.4	61.8
	DN2.0	72.2	57.9	62.2
	DN3.0	75.2	-	-

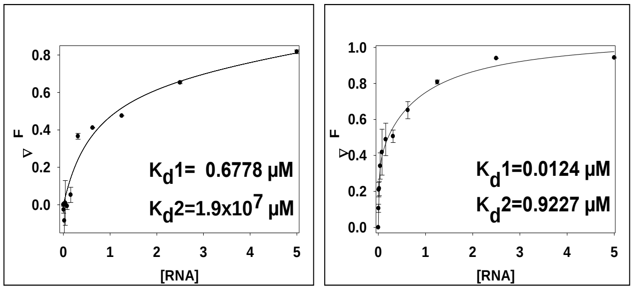

(5'CAG/3'GAC)×1 (5'CAG/3'GUC)×1

CP9

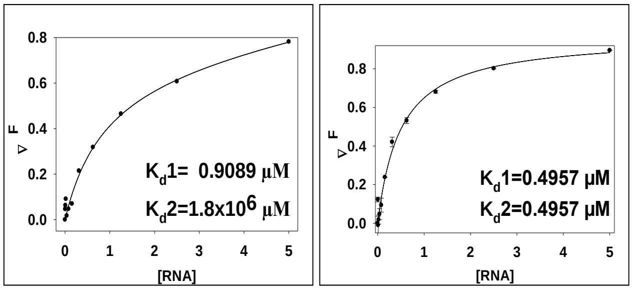

(5'CAG/3'GAC)×1 (5'CAG/3'GUC)×1

CP10

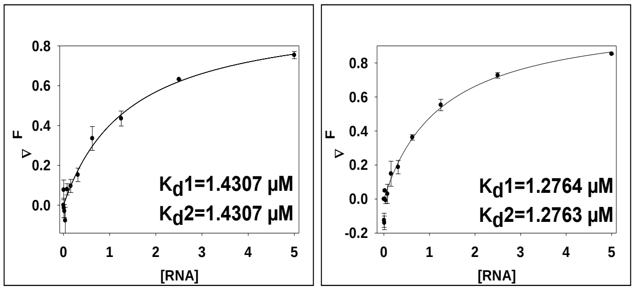

(5'CAG/3'GAC)×1 (5'CAG/3'GUC)×1

CP11

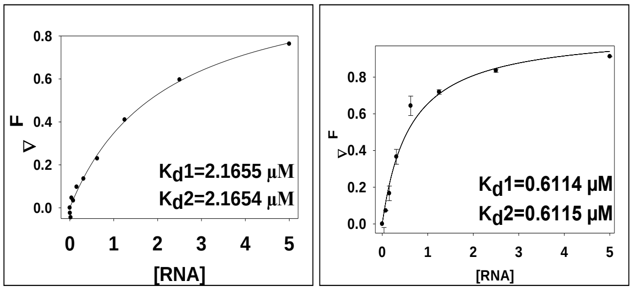

(5'CAG/3'GAC)×1 (5'CAG/3'GUC)×1

CP12

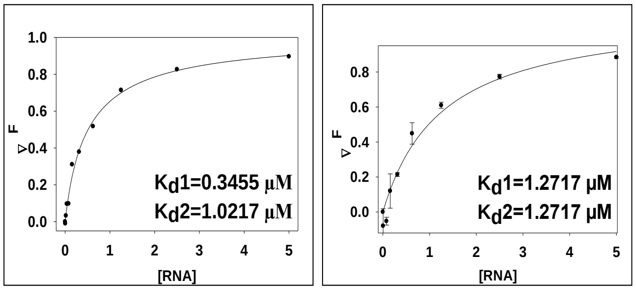

(5'CAG/3'GAC)×1 (5'CAG/3'GUC)×1

CP13

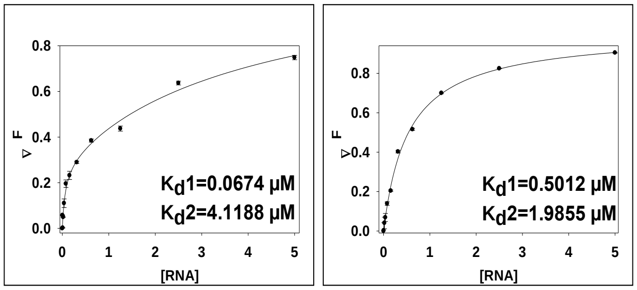

(5'CAG/3'GAC)×1 (5'CAG/3'GUC)×1

CP14

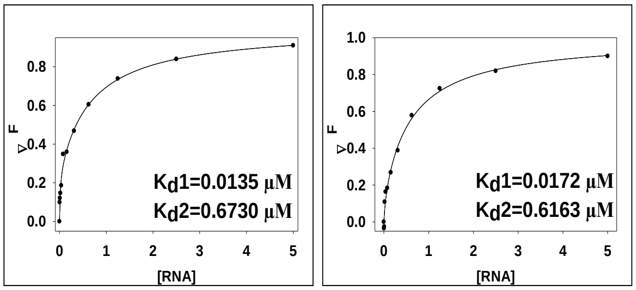

(5'CAG/3'GAC)×1 (5'CAG/3'GUC)×1

CP15

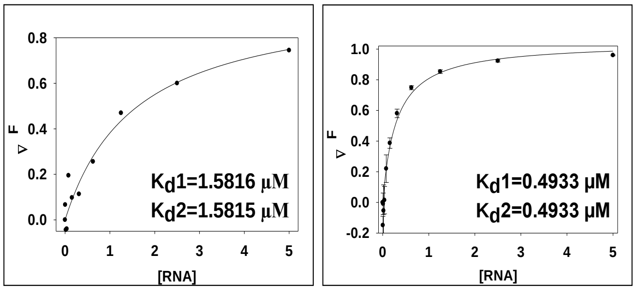

(5'CAG/3'GAC)×1 (5'CAG/3'GUC)×1

CP16

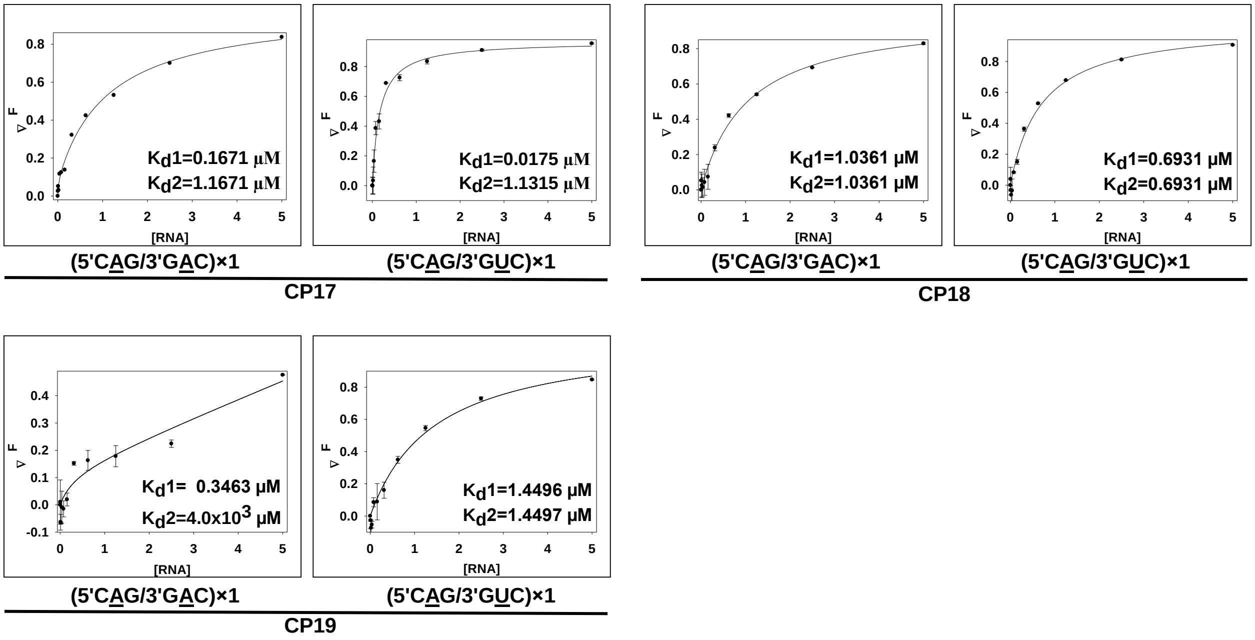

Figure S1. Fluorescence titration curves of RNAs (5'CAG/3'GAC)×1 & (5'CAG/3'GUC)×1 RNA respectively with different compounds. Solid lines represent the data fitting by two sites saturation ligand binding mode. Values of binding constant (s) (K_d) are mentioned in right bottom side of the plot.

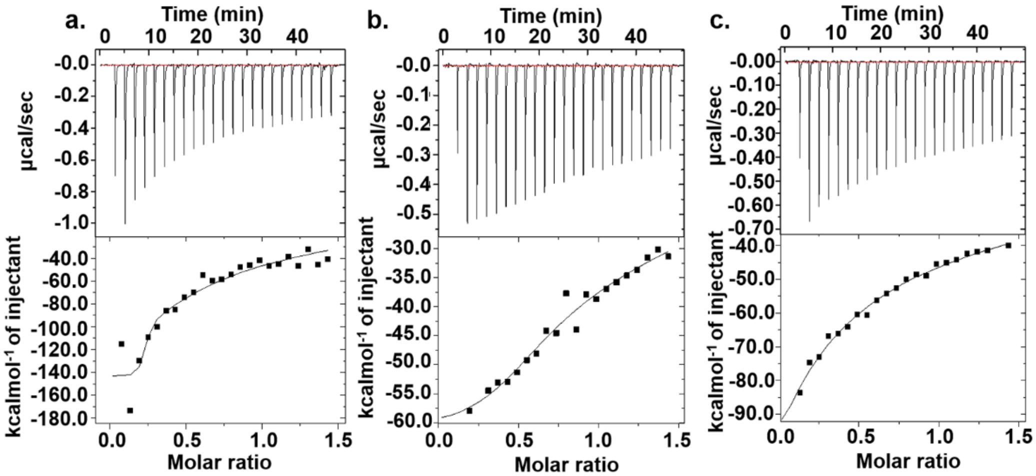

Figure S2. Isothermal titration calorimetry assay of (5′C**A**G/3′G**U**C)x6 RNA with compounds **(a)** CP2 **(b)** CP6 **(c)** CP13

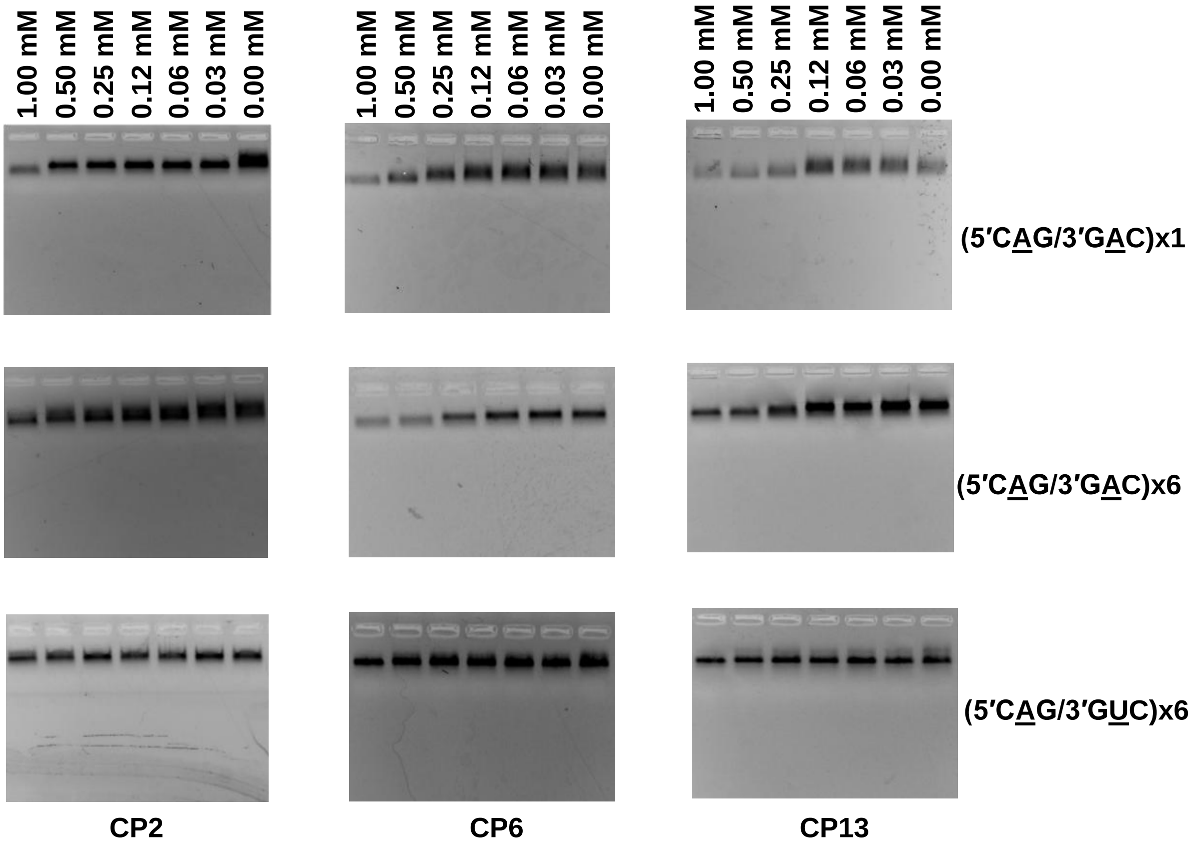

Figure S3a. PCR stop assay of compounds CP2, CP6 & CP13 **(Left to right)** with different motif DNA templates (5'C**A**G/3'G**A**C)x1, (5'C**A**G/3'G**A**C)x6 & (5'C**A**G/3'G**U**C)x6 respectively **(top to bottom).**

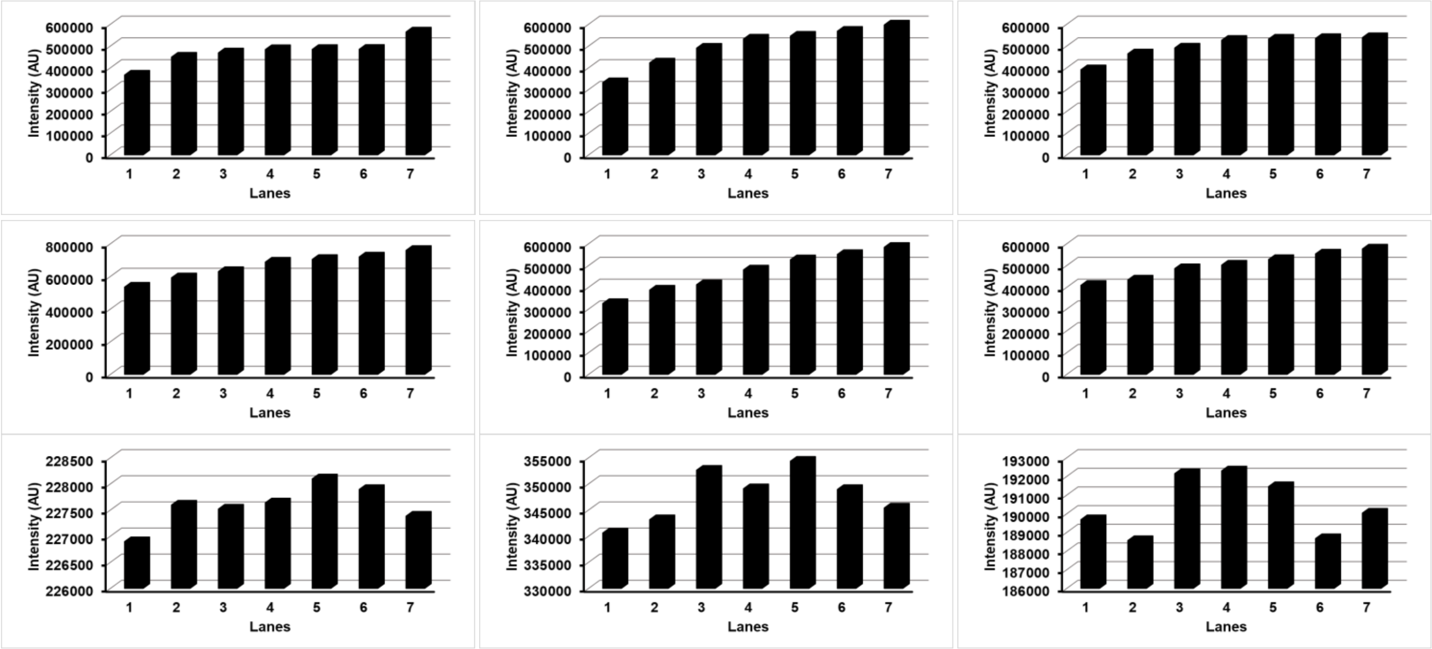

Figure S3b. Gel intensity calculated using ImageJ for PCR stop assay. Plots depicts the results for CP2, CP6 & CP13 **(Left to right)** with different motif DNA templates (5’C**A**G/3’G**A**C)x1, (5’C**A**G/3’G**A**C)x6 & (5’C**A**G/3’G**U**C)x6 respectively **(top to bottom).**

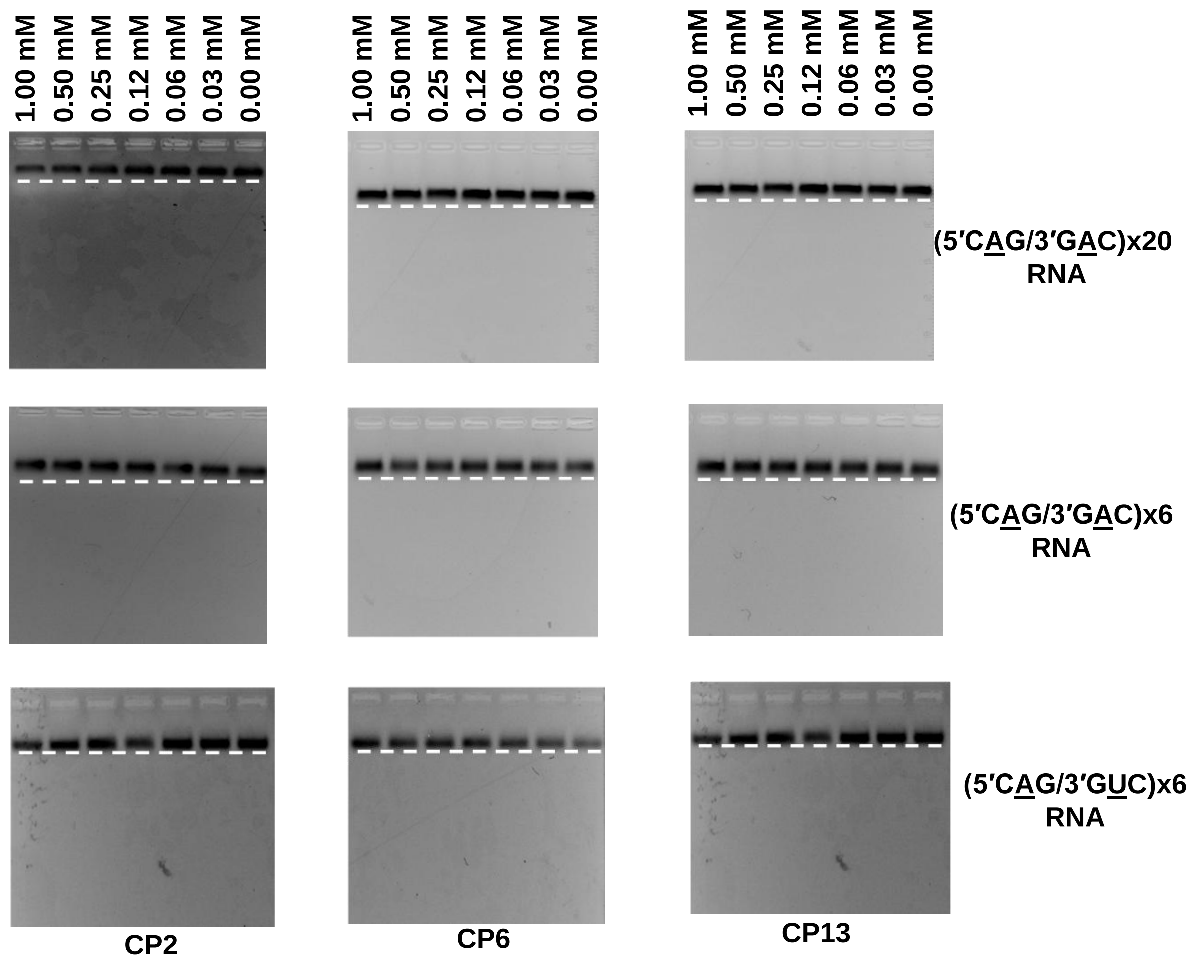

Figure S4a. Gel retardation assay of compounds CP2, CP6 & CP13 **(Left to right)** with different motif RNA templates (5'C**A**G/3'G**A**C)x20, (5'C**A**G/3'G**A**C)x6 & (5'C**A**G/3'G**U**C)x6 respectively **(top to bottom).**

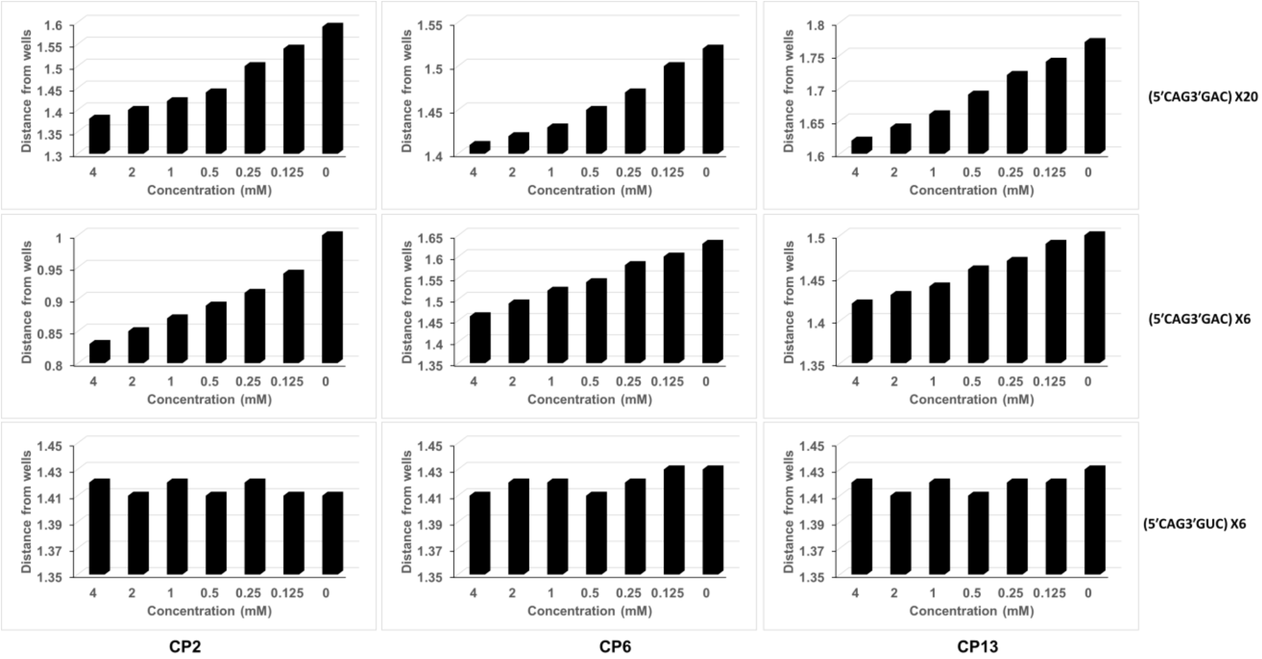

Figure S4b. Plots for distance travelled from wells in gel retardation assay of compounds CP2, CP6 & CP13 (Left to right) with different motif RNA templates (5’C**A**G/3’G**A**C)x20, (5’C**A**G/3’G**A**C)x6 & (5’C**A**G/3’G**U**C)x6 respectively (top to bottom).

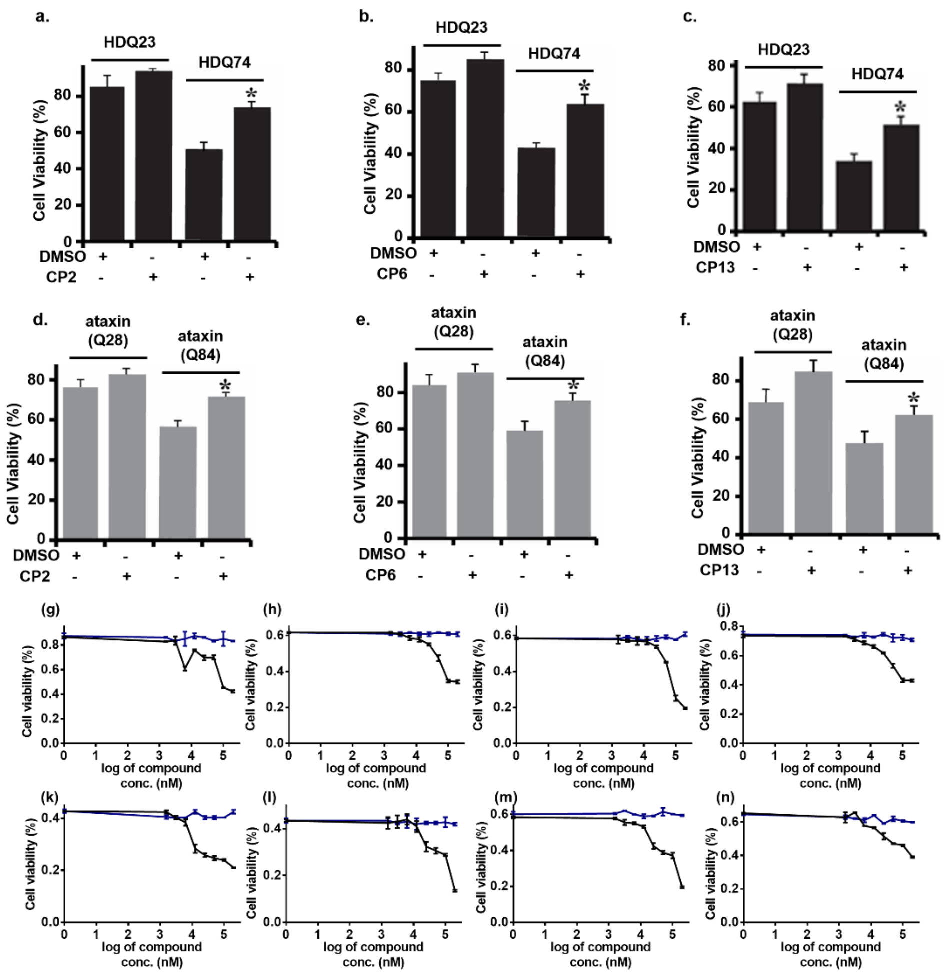

Figure S5. Cell viability assay in HD cellular models. **(a-f)** Cos-7 cells were transfected with polyglutamine encoding plasmids (HDQ23, HDQ74, ATX28 and ATX84) and treated with compounds to assess the cyto-protection effects of compounds. **(g-n)** Viability of cells treated with compounds on normal HEK293 cells with **(g)** CP2 **(h)** CP3 **(i)** CP4 **(j)** CP6 **(k)** CP8 **(l)** CP13 **(m)** CP14 **(n)** Myricetin. Blue line showing the curve for vehicle (control) while black lines represents viability of cells treated with compounds. Values shown are the mean ± SD from three independent sets of experiments. *p<0.05 compared to control

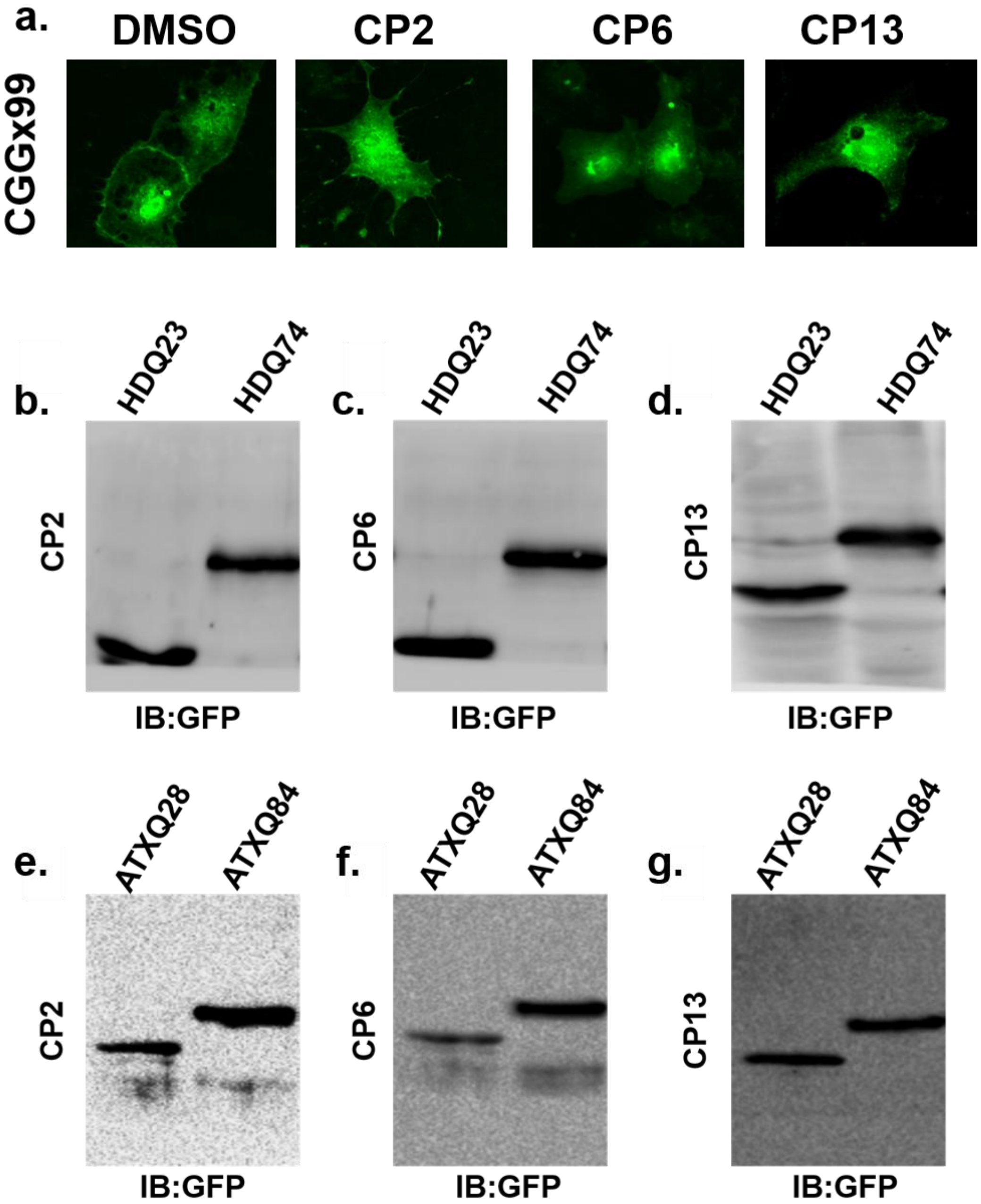

Figure S6 **(a)** Micrographs of EGFP-CGG99, transiently transfected COS-7 cells, treated with control (DMSO) as well as compounds (100.0 µM) for 12 hours **(b-g) Full images of western blots.** Plasmid expression analysis of the cells was confirmed by immunoblot, using anti-GFP antibodies **(top lane)** HDQ plasmid treated with **(a)** CP2 **(b)** CP6 **(c)** CP13 **(bottom lane)** ATX plasmid treated with **(d)** CP2 **(e)** CP6 **(f)** CP13
